# An In Vivo Electroencephalographic Analysis of the Effect of Riluzole against Limbic and Absence Seizure and Comparison with Glutamate Antagonists

**DOI:** 10.3390/pharmaceutics15072006

**Published:** 2023-07-22

**Authors:** Rita Citraro, Francesca Bosco, Gianfranco Di Gennaro, Martina Tallarico, Lorenza Guarnieri, Luca Gallelli, Vincenzo Rania, Antonio Siniscalchi, Giovambattista De Sarro, Antonio Leo

**Affiliations:** 1 Section of Pharmacology, Science of Health Department, School of Medicine, University “Magna Graecia” of Catanzaro, 88100 Catanzaro, Italy; 2 Research Center FAS@UMG, Department of Health Science, University “Magna Graecia” of Catanzaro, 88100 Catanzaro, Italy; 3 Department of Neurology and Stroke Unit, Annunziata Hospital of Cosenza, 87100 Cosenza, Italy

**Keywords:** excitatory neurotransmission, limbic seizure, absence epilepsy, glutamatergic antagonists, antiseizure medications, Riluzole

## Abstract

Background: Riluzole (RLZ) has demonstrated neuroprotective effects in several neurological disorders. These neuroprotective effects seem to be mainly due to its ability to inhibit the excitatory glutamatergic neurotransmission, acting on different targets located both at the presynaptic and postsynaptic levels. Methods: In the present study, we evaluated the effects of Riluzole (RLZ) against limbic seizures, induced by AMPA, kainate, and NMDA receptor agonists in Sprague–Dawley rats, and in a well-validated genetic model of absence epilepsy, the WAG/Rij rat. Furthermore, in this latter model, we also studied the effect of RLZ in co-administration with the competitive NMDA receptor antagonist, CPP, or the non-competitive AMPA receptor antagonist, THIQ-10c, on spike-wave discharges (SWDs) in WAG/Rij rats, to understand the potential involvement of AMPA and NMDA receptors in the anti-absence effect of RLZ. Results: In Sprague–Dawley rats, RLZ pretreatment significantly reduced the limbic seizure severity induced by glutamatergic agonists, suggesting an antagonism of RLZ mainly on NMDA rather than non-NMDA receptors. RLZ also reduced SWD parameters in WAG/Rij rats. Interestingly, the co-administration of RLZ with CPP did not increase the anti-absence activity of RLZ in this model, advocating a competitive effect on the NMDA receptor. In contrast, the co-administration of RLZ with THIQ-10c induced an additive effect against absence seizure in WAG/Rij rats. Conclusions: these results suggest that the antiepileptic effects of RLZ, in both seizure models, can be mainly due to the antagonism of the NMDA glutamatergic receptors.

## 1. Introduction

Riluzole (2-amino-6-trifluoromethoxy-benzothiazole (RLZ)) is the first FDA-approved medication against amyotrophic lateral sclerosis (ALS) [1], with neuroprotective properties also in other neurological diseases, such as brain ischemia, traumatic brain injury, Parkinson’s, and Alzheimer’s diseases [2,3,4]. These neuroprotective effects of RLZ seem to be mainly due to its ability to inhibit the excitatory glutamatergic neurotransmission, acting on different targets located both at the presynaptic and postsynaptic levels [5,6].

Besides this anti-glutamatergic effect, the properties of RLZ can also be secondary to the inhibition of Na^+^, K^+^, and Ca^2+^ channel activity and/or protein kinase C [7]. Similarly, it has also been documented that RLZ can also strengthen the inhibitory GABAergic neurotransmission [8,9]. As is widely reported, an imbalance between excitatory (E) and inhibitory (I) neurotransmission is strongly implicated in neuronal network hyperexcitability and spontaneous recurrent seizures (SRSs) onset [10]. This hypothesis supports a key role for glutamate-mediated neuronal hyperexcitation, having a causative role in producing seizure. In fact, an increase in glutamate levels in the extracellular fluid during seizure is well-documented [10,11]. To date, it is well-recognized that agonists of glutamatergic ionotropic receptors can provoke seizures in animal or human subjects, while antagonists have been shown to reduce seizures in preclinical models and patients with epilepsy (PWE), supporting the development of antiseizure medication targeting NMDA and AMPA receptors [11,12,13]. RLZ and its derivates, by recovering the altered ratio between E and I neurotransmission, can be used as monotherapy or adjunctive therapy in PWE [7]. Currently, different studies performed in experimental models of epilepsy have recognized the effectiveness of RLZ. Particularly, in vitro studies, RLZ was effective in reducing epileptiform activity in hippocampal slices [14,15,16] and primary cell culture [17]. Likewise, it was also shown that RLZ had antiseizure effects in various in vivo models of epilepsy, such as in the DBA/2 mouse model [18], in the pilocarpine model [19,20], in the maximal electroshock test [21], and in the amygdala kindled rats, where the drug delayed seizure appearance and reduced after discharge duration [22].

Based on this background, we aimed to investigate the RLZ effect against limbic convulsions induced by different glutamatergic receptor agonists (NMDA, AMPA, or kainate) in Sprague–Dawley rats. We also aimed to better understand the potential effect of RLZ, which is poorly understood, against absence seizure in the WAG/Rij rat model. The WAG/Rij rat model is a validated animal model of absence epilepsy, epileptogenesis, and neuropsychiatric comorbidities, which has also been used to document an involvement of the glutamatergic pathways in the initiation and maintenance of seizure [23,24]. To characterize the type of ionotropic glutamate receptor involved in the antiseizure property of RLZ, we also studied the effects of the co-administration of RLZ with the competitive NMDA receptor antagonist, CPP ((3-(2-carboxypiperazine-4-yl)-1-phosphonate)) or the non-competitive AMPA receptor antagonist THIQ-10c (N-Acetyl-1-(4-chlorophenyl)-6,7-dimethoxy-1,2,3,4-tetrahydroisoquinoline).

## 2. Materials and Methods

### 2.1. Animals

Experiments were performed in male Sprague–Dawley ((SD) 9- to 11-week-old) and WAG/Rij rats (6 months of age) purchased from Charles River Laboratories s.r.l. (Calco, Lecco, Italy). Rats were housed 3 or 4 per cage and maintained under controlled environmental conditions (60 ± 5% humidity; 22 ± 2 °C; 12/12 h reversed light/dark cycle; lights on at 20.00). Standard laboratory chow and tap water were provided ad libitum. All procedures involving animals and their care were accomplished in agreement with the international and national laws and policies (EU Directive 2010/63/EU for animal experiments, ARRIVE guidelines, and the Basel declaration, including the 3R concept). The experimental protocols and the procedures described were approved (Authorization n° 320/2019-PR) by the Animal Care Committee of the University of Catanzaro, Italy. All efforts were made to reduce animal suffering and reduce the number of animals used.

### 2.2. Drugs

RLZ (Merck, Milan, Italy) was dissolved in 10% DMSO and 90% sterile saline. NMDA, AMPA and kainate were purchased from Tocris (Buckhurst Hill, UK). The doses of glutamate receptor agonists (NMDA, AMPA) were dissolved in saline and delivered in a volume of 2 µL by a Hamilton syringe connected to a CMA/100 infusion pump. The competitive NMDA receptors antagonist, (±) 3-(2-carboxypiperazine-4-yl)-1-phosphonate) (CPP), was purchased from Merck and dissolved in normal saline solution to obtain the desired concentrations. The non-competitive AMPA receptor antagonist, N-acetyl-1-(4-chlorophenyl)-6,7-dimethoxy-1,2,3,4-tetrahydroisoquinoline (THIQ-10c), was synthesized by Prof. Alba Chimirri’s group (Department of Medicinal Chemistry, University of Messina, Messina, Italy). THIQ-10c was dissolved in a 40% 2-hydroxypropyl-γ-cyclodextrin with sonication and mild heating.

### 2.3. Experimental Protocol in Sprague–Dawley Rats

**Figure 1:**
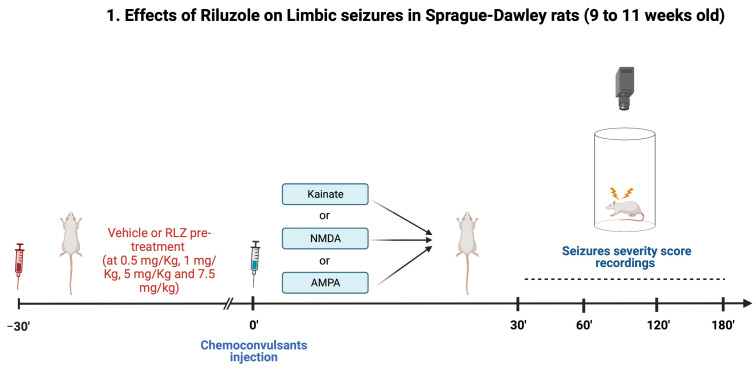
Experimental design in Sprague–Dawley rats. Created by BioRender.com (accessed on 3 April 2023).

We planned two different experiments in SD rats (Figure 1); in the first, we aimed to evaluate the effect of RLZ on KA-induced seizures, SD rats were randomly subdivided into the following five groups (n = 8 for each group):

(1) vehicle + KA;
(2) RLZ 0.5 mg/kg + KA;
(3) RLZ 1 mg/kg + KA;
(4) RLZ 5 mg/kg + KA;
(5) RLZ 7.5 mg/kg + KA.

RLZ (0.5, 1, 5 and 7.5 mg/kg; i.p.) was administered 30 min prior to seizure induction in SD rats. Seizures were induced by injection of KA (12 mg/kg; i.p.), which was dissolved in distilled water. The choice of KA dose was based on a previous study [13]. After the KA injection, rats were placed individually in a plexiglas box and observed for 3 h for the development of seizures. Seizure severity score was rated by the scale of Racine et al. 1972: stage 1, facial clonus; stage 2, wet dog shakes and head nodding; stage 3, forelimb clonus; stage 4, forelimb clonus with rearing; stage 5, rearing, jumping and falling [25]. Once SD rats achieved stages 4 and/or 5, seizures were observed for up to 60 min and then ended with diazepam (10 mg/kg) to reduce the mortality rate. Seizures were always ended by diazepam after 3 h [26].

In the second, we also planned to study the effect of different doses of RLZ against AMPA- or NMDA-evoked convulsions in a separate set of SD rats. Because of this, SD rats were randomly divided into the following 5 groups (n = 8; for each group) for convulsant (AMPA or NMDA) administered:

(1) vehicle + AMPA or NMDA;
(2) RLZ 0.5 mg/kg + AMPA or NMDA;
(3) RLZ 1 mg/kg + AMPA or NMDA;
(4) RLZ 5 mg/kg + AMPA or NMDA;
(5) RLZ 7.5 mg/kg + AMPA or NMDA.

Subsequently, SD rats were chronically implanted, under general anesthesia (tiletamine/zolazepam 1:1; Zoletil 100^®^; 50 mg/kg i.p.; VIRBAC Srl, Milan, Italy), with a guide cannula for intracerebroventricular (i.c.v.) administration (from Bregma. AP = −1.3; L = 0; H = 4.5) as previously described [27]. The i.c.v. administration of AMPA (10 nmol/2 μL) or NMDA (10 nmol/2 μL) was made using a 5 μL Hamilton microsyringe connected to a CMA/100 infusion pump. The injection cannulae was lowered 2 mm beyond the edge of the guide cannula and withdrawn 1 min following infusion [18,28]. RLZ (0.5, 1, 5, and 7.5 mg/kg) was i.p. administered 30 min before i.c.v. administration of AMPA or NMDA. The intensity of seizures induced by AMPA or NMDA was assessed using the following scoring system: 0, no response; 1, myoclonic jerks of forelimb; 2, mouth and facial movements (i.e., facial myoclonus, clonus of the jaw and vibrissae) and head nodding with or without mild forelimb clonus; 3, severe forelimb clonus; 4, rearing and falling down, hindlimb clonus and forelimb tonus; and 5, tonic extension of the hindlimb, status epilepticus, and/or death. The maximum response was recorded for each rat, as previously described by De Sarro et al. [18,28].

### 2.4. Experimental Protocol in WAG/Rij Rats

We planned two different experiments in WAG/Rij rat (n = 136) model, both to study the RLZ effect, intraperitoneally or focally administered, against absence seizures and to understand the potential involvement of AMPA and NMDA receptors in its potential effect. To this latter point, RLZ was co-administered with CPP, a NMDA receptor antagonist, and THIQ-10c, an AMPA receptor antagonist.

**Figure 2:**
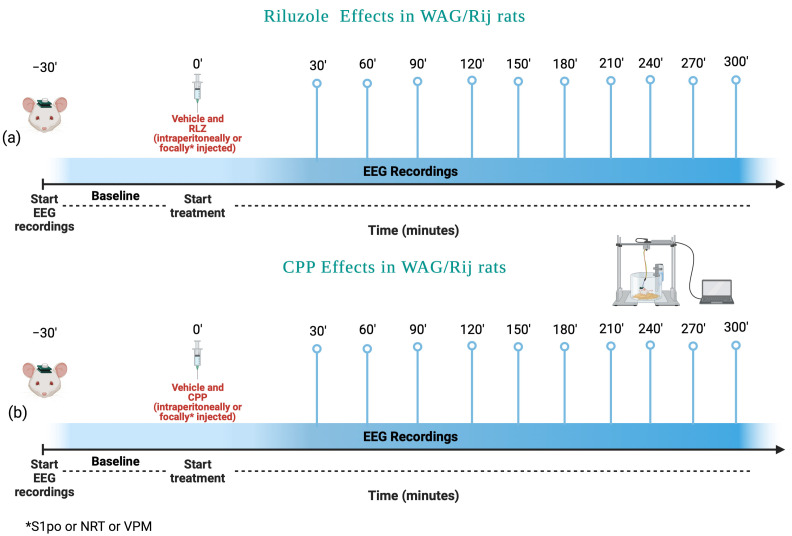
Experimental scheme used to evaluate the effects of (**a**) Riluzole and (**b**) CPP in WAG/Rij rats. Created by BioRender.com (accessed on 3 April 2023).

RLZ was administered either i.p. or focally, by bilateral microinjections, both into some selected thalamic nuclei of the ventrobasal complex, including the nucleus ventralis posteromedialis thalami (VPM), nucleus reticularis thalami (NRT), and into the peri-oral region of the primary somatosensory cortex (S1po). These brain areas were selected being those essential for the generation and maintenance of absence seizures [23]. Male WAG/Rij rats, at 6 months of age, were randomly divided into two groups for the route of RLZ administration. The first group of WAG/Rij rats was randomly divided into the following five subgroups (n = 8 for each dose and vehicle; Figure 2a):

(1) Vehicle;
(2) RLZ 0.5 mg/kg;
(3) RLZ 1 mg/kg;
(4) RLZ 5 mg/kg;
(5) RLZ 7.5 mg/kg.

The second group of rats was focally injected after being randomly divided into three subgroups for the selected brain area (VPM, NRT, and S1po). Each of these subgroups was further divided into the following four groups (n = 8 for each dose and vehicle; Figure 2a):

(1) Vehicle;
(2) RLZ 0.25 μg/0.5 μL;
(3) RLZ 0.5 μg/0.5 μL;
(4) RLZ 1 μg/0.5 μL.

We aimed to evaluate the effects of RLZ, at 7.5 mg/kg/i.p., in co-administration with CPP, an NMDA receptor antagonist or THIQ-10c, an AMPA receptor antagonist, administered either i.p. or focally microinjected into the S1po region. First, we performed a dose-finding study in adult WAG/Rij rats to select the optimal therapeutic dose of CPP for the coadministration studies (Figure 2b). With this aim, male WAG/Rij rats were randomly divided into two groups for each route of administration used. The first group, after being randomly divided into the following four subgroups (n = 8 for each dose), was used to study the effects of intraperitoneal administrations of CPP on absence seizures.

(1) Vehicle;
(2) CPP 5 mg/kg;
(3) CPP 10 mg/kg;
(4) CPP 15 mg/kg.

The second group, after being randomly divided into the following four subgroups (n = 8 for each dose), was used to study the effects of focal microinjections of CPP on absence seizures.

(1) Vehicle;
(2) CPP 5 pmol/μL;
(3) CPP 10 pmol/μL;
(4) CPP 20 pmol/μL.

The optimal therapeutic dose of THIQ-10c was chosen in agreement with previously published studies [29,30].

**Figure 3:**
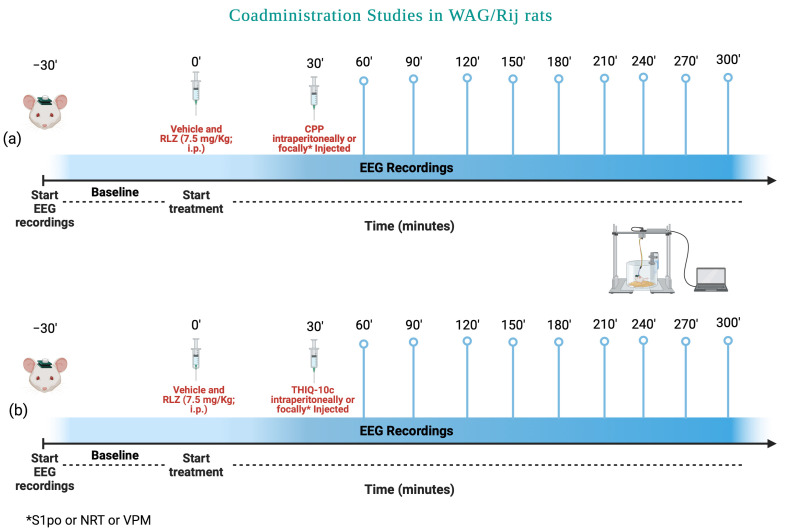
Experimental scheme used to evaluate the co-administration effects of Riluzole with (**a**) CPP and (**b**) THIQ-10c in WAG/Rij rats. Created by BioRender.com (accessed on 3 April 2023).

Subsequently, a different set of WAG/Rij rats, at 6 months of age, was used for the coadministration study (Figure 3). These rats were randomly divided into two different subgroups for each antagonist used. The first subgroup of each experimental group was used to test both the effects of CPP and THIQ-10c, focally microinjected at 20 pmol/μL and 20 mg/μL, respectively, with RLZ at 7.5 mg/kg/i.p.

The second subgroup of each experimental group was used to test both the effects of CPP and THIQ-10c at 15 mg/kg i.p. and 10 mg/kg i.p., respectively, with RLZ at 7.5 mg/kg/i.p. Identical matched WAG/Rij rat vehicle control subgroups were included in the study.

#### Animal Surgery and EEG Recording

Adult WAG/Rij rats were chronically implanted, under general anesthesia (tiletamine/zolazepam 1:1; Zoletil 100^®^; 50 mg/kg i.p.; VIRBAC Srl, Milan, Italy), with 3 cortical electrodes fastened to a 3-channel rat headmount (8239-SE3; Pinnacle Technology, Stoke-on-Trent, UK). Three stainless-steel screw electrodes were implanted on the dura mater over the cortex: two in the frontal region (AP = −2; L = ±2.5) and the ground/reference electrode over the cerebellum [31].

For focal administration of RLZ, guide cannulae were bilaterally placed in the following brain areas primarily involved in the generation of absence seizures [29]: nucleus ventralis posteromedialis thalami (VPM, AP = −3.3; L = ±2.6; H = 6 mm from bregma), nucleus reticularis thalami (NRT, AP = −2.8; L = ±3.7; H= 5.4 mm from bregma), and into the peri-oral region of the primary somatosensory cortex (S1po, AP = −2.1; L = ±5.5; H = 4 mm from bregma) according to the coordinates of the atlas by [32]. For the focal administration of RLZ, rats were gently hand-restrained, and drug infusions were made bilaterally in a volume of 0.5 μL/side, using injector cannulae connected by a polyethylene tube to a Hamilton syringe, as previously described [29]. We also examined the effects of the co-administration of RLZ with NMDA or AMPA receptor antagonists CPP and THIQ-10c, injected i.p. or in the S1po cortex. After surgery, rats were allowed at least 7 days of recovery and then connected to preamplifiers (Pinnacle Technology’s 8400–9000 video/EEG system with Sirenia Seizure PRO 1.8.4 Software (Lawrence, KS, USA)), for at least 3 days before the experiments to habituate animals to the recording procedures. To avoid circadian alterations within groups, every EEG session started at 9:00 am. EEG signals were amplified and conditioned by analog filters in accordance with previous experiments [27,33]. For single administration, EEG recordings lasted for 5 h after treatment. For co-administration, EEG recording sessions comprised 30 min of baseline without injection, 30 min of pretreatment with RLZ, and 4 h after the injection of antagonist (CPP or THIQ-10c). Quantification of epileptic seizures, performed by two trained investigators blinded for the experimental protocol, was based on the number and the duration of EEG SWDs according to well-established criteria. Briefly, the number and duration of SWDs for each rat were summarized in 30 min intervals (epochs) and counted by visual inspection of the EEG recordings; all recordings were evaluated by two independent researchers who were blinded to the treatment. An SWD was considered as an EEG background deflection characterized by a 7.5–9.5 Hz frequency and an amplitude at least double that of the background with a minimal duration of 1 s. To study the effects of treatments, every recording session was separated into 30 min epochs; the cumulative SWD duration and number per epoch were computed and presented as means ± standard error of the mean (SEM) [31,34].

### 2.5. Statistical Analysis

Severity, duration, and number of crises were summarized using mean and standard error of the mean (SEM) and stratifying by dose and time point. Severity was treated as an ordinal variable and modeled using ordered logistic regression, introducing dose, time, and their interaction as predictors. Pairwise comparisons between all doses at each of the 4 time points were performed. The number and duration of crises were modeled using a restricted cubic splines model with six knots. This approach was employed to account for the non-linearity of the temporal trajectories. The knots were evenly distributed and aligned with the 5th, 23rd, 41st, 59th, 77th, and 95th percentiles of the time variable, as suggested in the literature [35]. The spline–dose interaction was introduced in the model, together with the baseline value. Pairwise comparisons between doses were performed at the most significant time points, namely 30′, 60′, 90′, 120′, and 180′. In each model, a random intercept was introduced to account for the within-subject correlation of the measurements. Statistical significance was set at 5%. All pairwise comparisons were adjusted for statistical significance using the Bonferroni correction method. No pre-planned missing data treatment was conducted. The normality of residuals was assessed for each model by standardized normal probability plots. The statistical analyses were performed using STATA.com ((accessed on 6 July 2023) software, version 17. All details regarding regression models and between doses pairwise comparisons at each timepoint are provided in the Supplementary Materials.

## 3. Results

### 3.1. Effects of RLZ on KA-Induced Seizure in Sprague–Dawley Rats

**Figure 4:**
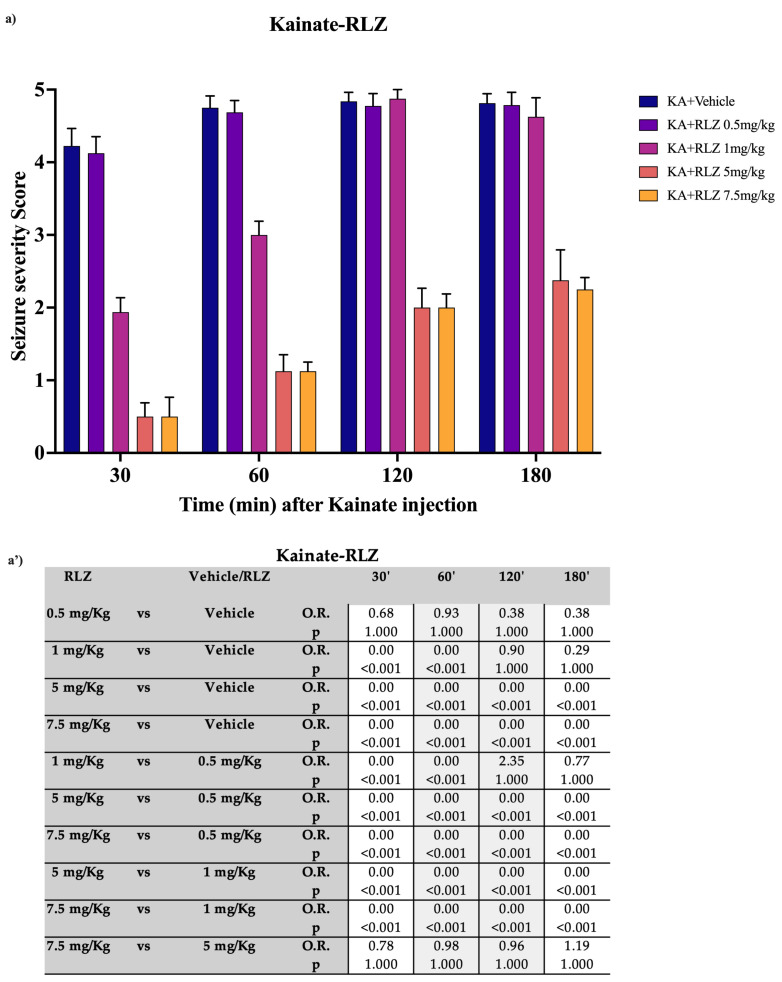
Effect of RLZ pretreatment against kainate-induced seizure in SD rats. (**a**) Bars indicate the seizure severity score. Data are expressed as means ± standard error of the mean (SEM) of 8 animals for each dose. (**a’**) Table reports the pairwise comparisons between doses. Bonferroni correction was performed. KA: kainate; RLZ: Riluzole.

Consistent with other studies, the systemic administration of KA to adult SD rats resulted in well-characterized complex seizure activity starting within 30 min, with head nodding and wet dog shakes, which evolved into severe limbic seizures [13,36]. As depicted in Figure 4a,a’, the severity scores exhibit lower values at 30 min and progressively increase until reaching 180 min, indicating a presumed decline in the drug’s efficacy. Upon evaluating pairwise comparisons, it was observed that the dose of 0.5 mg/kg at 30 min did not demonstrate a lower odds of severity score compared with the vehicle (OR: 0.68; 95% CI: 0.01, 31.47). In contrast, the doses of 1, 5, and 7.5 mg/kg at 30 min were strongly associated with lower severity scores relative to the vehicle, with odds ratios (ORs) approaching zero. Dose 7.5 mg/kg and dose 5 mg/kg exhibited comparable efficacy without significant differences (OR: 0.78; 95% CI: 0.01, 53.79), while dose 5 mg/kg was more effective than dose 0.5 mg/kg (OR: 0.00; 95% CI: 0.00, 0.54), and dose 1 mg/kg was more effective than the lower dose (OR: 0.00; 95% CI: 0.00, 0.24). In subsequent timepoints, along with the overall decline in efficacy, no statistically significant differences were observed between the two higher doses.

### 3.2. Effects of RLZ on Seizure NMDA or AMPA-Induced in Sprague–Dawley Rats

**Figure 5:**
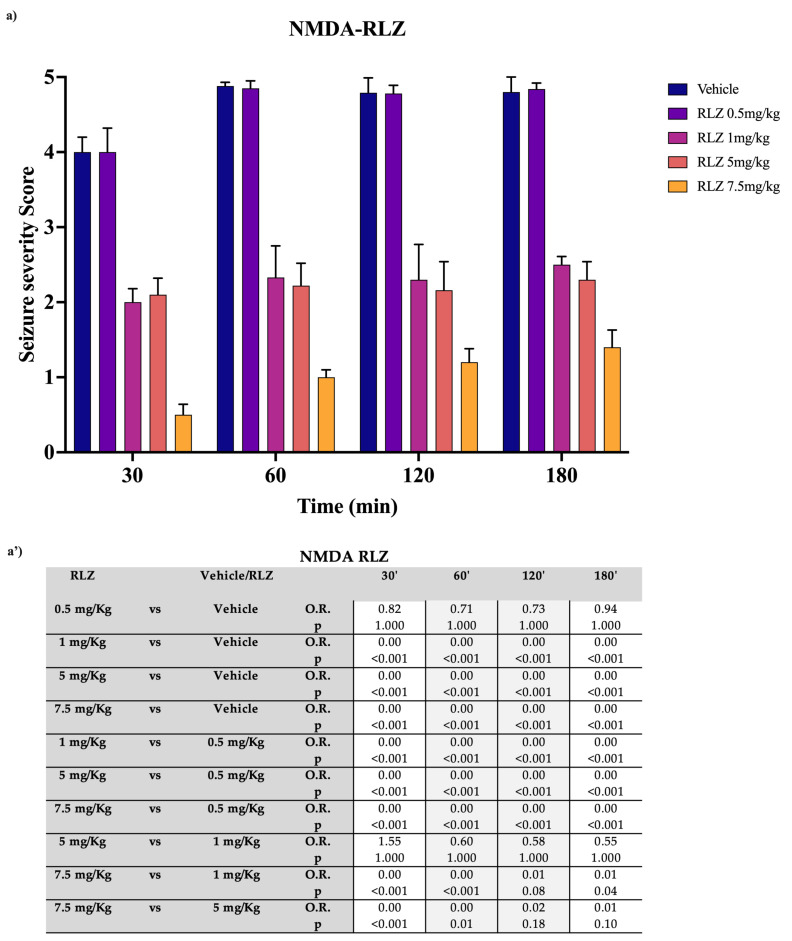
Effect of RLZ pretreatment against NMDA-induced seizure in SD rats. (**a**) Bars indicate the seizure severity score. Data are expressed as means ± standard error of the mean (SEM) of 8 animals for each dose. (**a’**) Table reports the pairwise comparisons between doses. Bonferroni correction was performed. KA: kainate; RLZ: Riluzole.

It has also been demonstrated that i.c.v. injections of NMDA can produce generalized seizures [37]. The first symptom is an increased locomotor activity tremor and head-bobbing, hypermotility that preceded the first clonic episode. Following these behavioral changes, the tonic component of the seizures occurred. As for kainate, the 0.5 mg/kg dose showed no efficacy compared with vehicle at any time point (Figure 5a,a’). On the contrary, the maximum efficacy, compared with the vehicle, was reached with a dose of 7.5 mg/kg at 30 min (OR: 0.00; *p* < 0.001). The 5 mg/kg dose at 30′ showed a significantly lower efficacy than the 7.5 mg/kg dose (OR: 0.00; *p* < 0.001) but not higher than that of the 1 mg/kg dose (OR: 1.55; *p* = 0.779). Moreover, from 60 min onwards, the 7.5 mg/kg dose progressively lost efficacy while remaining more effective than the vehicle, whereas the 5 mg/kg and 1 mg/kg doses did not undergo a noticeable decrease in efficacy.

**Figure 6:**
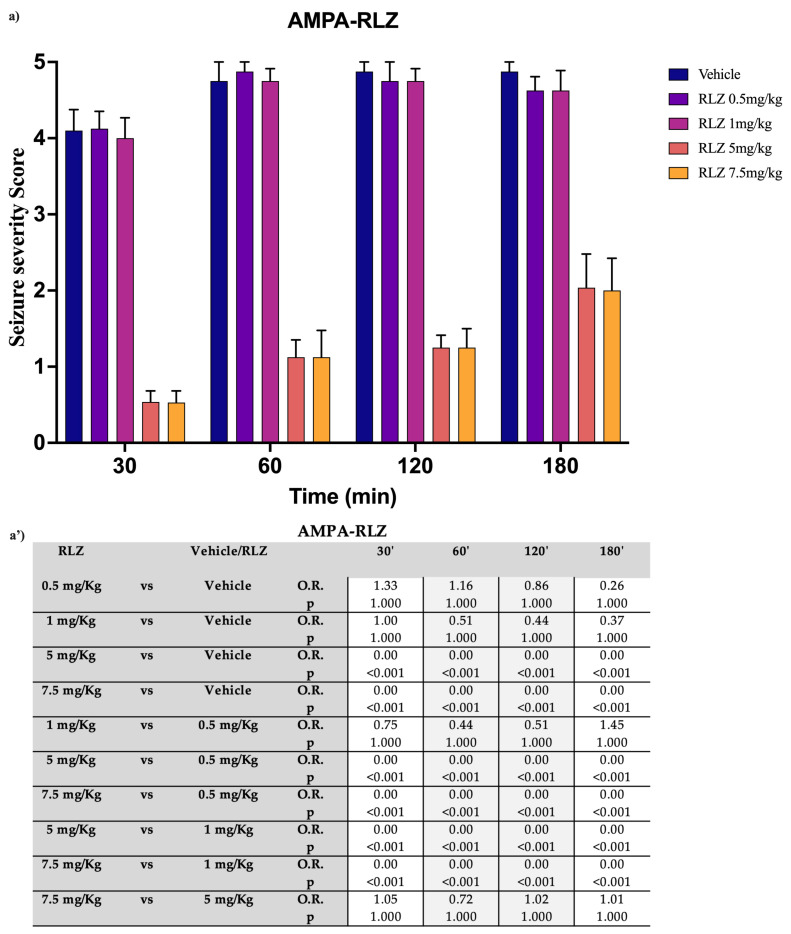
Effect of RLZ pretreatment against AMPA-induced seizure in SD rats. (**a**) Bars indicate the seizure severity score. Data are expressed as means ± standard error of the mean (SEM) of 8 animals for each dose. (**a’**) Table reports the pairwise comparisons between doses. Bonferroni correction was performed. KA: kainate; RLZ: Riluzole.

I.c.v. administration of AMPA induced generalized seizures similar to those caused by NMDA injections [37]. Regarding AMPA, the 0.5 mg/kg and 1 mg/kg doses did not show significant differences compared with the vehicle. On the contrary, the 5 mg/kg and 7.5 mg/kg doses exhibited peak efficacy at 30 min, with both doses significantly different from the vehicle (OR: 0.00; *p* < 0.001). The 7.5 mg/kg dose did not show superior efficacy compared with the 5 mg/kg dose at 30 min (OR: 1.04; *p* = 1.000) as in the other time points. Both doses equally lost efficacy from 60 min onwards (Figure 6a,a’).

### 3.3. Effects of RLZ, i.p. and Focally Administered on Absence Seizures in WAG/Rij Rats

**Figure 7:**
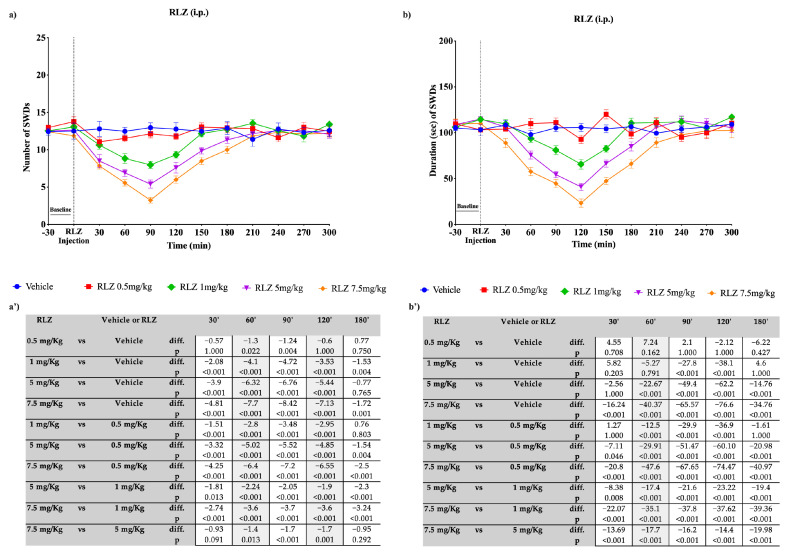
Effects of the i.p. administration of different doses of RLZ on SWDs in WAG/Rij rats. Plots show the effects of RLZ, at the four doses used, on the number (**a**) and duration (**b**) of SDWs in WAG/Rij rats. Tables report the pairwise comparisons between doses on the number (**a’**) and duration (**b’**) of SDWs in WAG/Rij rats, performed at the most significant time points, namely 30′, 60′, 90′, 120′, and 180′. Bonferroni correction was performed. Data are expressed as means ± standard error of the mean (SEM) of 8 animals for each dose. i.p.: intraperitoneal; RLZ: Riluzole; SWDs: spike-wave discharges.

The i.p. administration of RLZ (0.5, 1, 5, and 7.5 mg/kg) produced a significant dose-dependent reduction in absence seizures in WAG/Rij rats (Figure 7a,b), confirming the results by Romettino et al. [38].

The dose of 0.5 mg/kg was only able to significantly reduce the nSWDs at 60′ and 90′ compared with the vehicle. However, according to the small effect size (Figure 7a’), this effect can be considered not clinically significant. On the contrary, regarding the doses of 1 mg/kg, 5 mg/kg, and 7.5 mg/kg, the number of seizures progressively decreased from time 0 onwards, reaching the maximum reduction at 90 min. The maximum effectiveness was observed with the dose of 7.5 mg/kg (−8.42 seizures compared with the vehicle; *p* < 0.001), while the doses of 5 mg/kg and 1 mg/kg showed reductions of 6.76 (*p* < 0.001) and 4.72 (*p* < 0.001) seizures, respectively, compared with the vehicle. Regarding the duration of seizures, the peak effectiveness was observed at 120 min, where the doses of 7.5 mg/kg, 5 mg/kg, and 1 mg/kg showed a significant reduction (*p* < 0.001) in duration, with respective reductions of 65.57, 49.4, and 27.8 s compared with the vehicle. The dose of 0.5 mg/kg did not show effectiveness at any time point. Pairwise comparisons are reported in Figure 7a’,b’.

**Figure 8:**
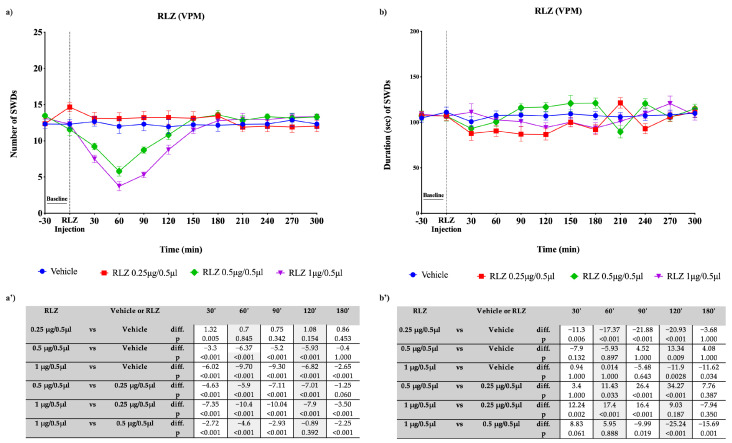
Effects of focal administration, into VPM, of different doses of RLZ on SWDs in WAG/Rij rats. Plots show the effects of RLZ at the three doses used and vehicle on the number (**a**) and duration (**b**) of SDWs in WAG/Rij rats. Tables report the pairwise comparisons between doses on the number (**a’**) and duration (**b’**) of SDWs in WAG/Rij rats, performed at the most significant time points, namely 30′, 60′, 90′, 120′, and 180′. Bonferroni correction was performed. Data are expressed as means ± standard error of the mean (SEM) of 8 animals for each dose. i.p.: intraperitoneal; RLZ: Riluzole; SWDs: spike-wave discharges; VPM: ventroposteromedial nucleus.

**Figure 9:**
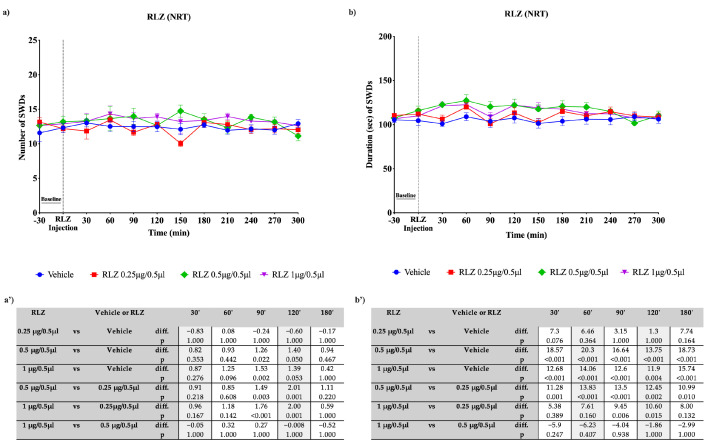
Effects of focal administration, into NRT, of different doses of RLZ on SWDs in WAG/Rij rats. Plots show the effects of RLZ, at the three doses used, and vehicle on the number (**a**) and duration (**b**) of SDWs in WAG/Rij rats. Tables report the pairwise comparisons between doses on the number (**a’**) and duration (**b’**) of SDWs in WAG/Rij rats, performed at the most significant time points, namely 30′, 60′, 90′, 120′, and 180′. Bonferroni correction was performed. Data are expressed as means ± standard error of the mean (SEM) of 8 animals for each dose. i.p.: intraperitoneal; NRT: nucleus reticularis thalami; RLZ: Riluzole; SWDs: spike-wave discharges.

**Figure 10:**
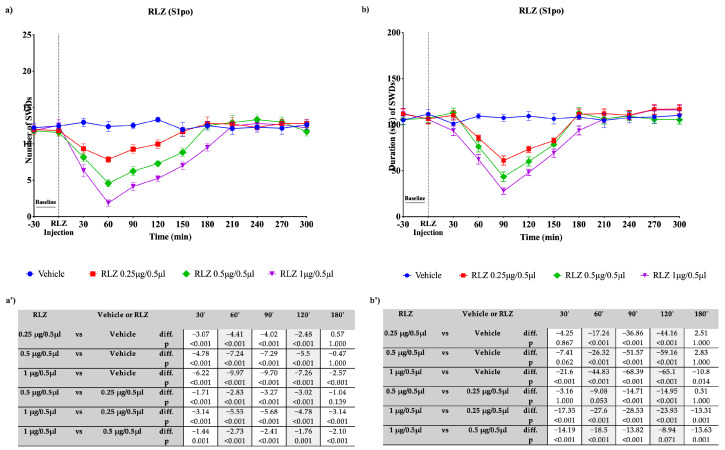
Effects of focal administration, into S1po, of different doses of RLZ on SWDs in WAG/Rij rats. Plots show the effects of RLZ, at the three doses used, and vehicle on the number (**a**) and duration (**b**) of SDWs in WAG/Rij rats. Tables report the pairwise comparisons between doses on the number (**a’**) and duration (**b’**) of SDWs in WAG/Rij rats, performed at the most significant time points, namely 30′, 60′, 90′, 120′, and 180′. Bonferroni correction was performed. Data are expressed as means ± standard error of the mean (SEM) of 8 animals for each dose. i.p.: intraperitoneal; RLZ: Riluzole; S1po: primary somatosensory cortex; SWDs: spike-wave discharges; VPM: ventroposteromedial nucleus.

The focal bilateral injection of RLZ (0.25, 0.5 and 1 μg/0.5 µL) was able to change the number and duration of SWDs compared with the vehicle (control) group depending on the site of administration. The focal bilateral injection of RLZ into the VPM induced a significant decrease (*p* < 0.001) in the nSWDs only at the two highest doses used: namely 0.5 and 1 μg/0.5 µL (Figure 8a,b). The maximum effectiveness was observed with the dose of 1 μg/0.5 µL (−9.70 seizures compared with the vehicle; *p* < 0.001) at 60′, while the dose of 0.5 μg/0.5 µL showed reduction of 6.37 (*p* < 0.001) seizures compared with the vehicle (Figure 8a’). Moreover, a statistical significance was also observed after RLZ injection into the VPM, at the three doses used in the dSWDs in WAG/Rij rats. However, according to the small effect size reported in Figure 8b’, this effect can be considered not clinically significant. Similarly, the focal bilateral injection of RLZ, at each dose used, into the NRT produced a significant difference in the nSWDs and dSWDs. However, according to the small effect size reported (Figure 9a’,b’), this can be considered not clinically significant. By contrast, the focal bilateral injection of RLZ (0.25, 0.5 and 1 μg/0.5 µL) into the S1po induced a significant (*p* < 0.001) dose-dependent decrease in the nSWDs and dSWDs, at several time points studied. The decrease in nSWDs appeared more clear (−9.97 seizures compared with the vehicle; *p* < 0.001) with the higher dose used, between 30 and 180 min after administration, with a peak at 60 min (Figure 10a,a’). Likewise, the decrease in dSWDs appeared more clear (−68.39 s compared with the vehicle; *p* < 0.001) with the higher dose used, between 30 and 180 min after administration, with a peak at 90 min (Figure 10b,b’).

### 3.4. Effects of CPP on Absence Seizures in WAG/Rij Rats

**Figure 11:**
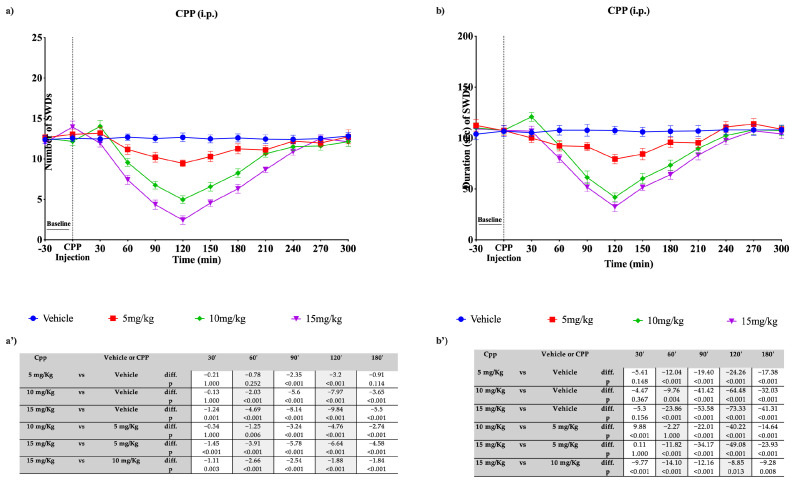
Effects of different doses of CPP, i.p. administered, on SWDs in WAG/Rij rats. Plots show the effects of CPP, at the three doses used, and vehicle on the number (**a**) and duration (**b**) of SDWs in WAG/Rij rats. Tables report the pairwise comparisons between doses on the number (**a’**) and duration (**b’**) of SDWs in WAG/Rij rats, performed at the most significant time points, namely 30′, 60′, 90′, 120′, and 180′. Bonferroni correction was performed. Data are expressed as means ± standard error of the mean (SEM) of 8 animals for each dose. CPP = 3-(2-carboxypiperazine-4-yl)-1-phosphonate; i.p.: intraperitoneal; SWDs: spike-wave discharges.

CPP (5, 10, and 15 mg/kg), a competitive NMDA receptor antagonist, was i.p. administered in WAG/Rij rats. Both the intraperitoneal and focal administration of CPP markedly reduced SWD parameters in a dose-dependent manner in WAG/Rij rats, as also displayed in other preclinical models of epilepsy [39,40]. In detail, regarding the doses of 5 mg/kg, 10 mg/kg, and 15 mg/kg intraperitoneally injected, the SWD parameters progressively decreased from time 0 onwards, reaching the maximum reduction at 120 min. Regarding nSWDs, the maximum effectiveness was observed with the dose of 15 mg/kg (−9.84 seizures compared with the vehicle; *p* < 0.001), while the doses of 10 mg/kg and 5 mg/kg showed reductions of 7.97 (*p* < 0.001) and 3.2 (*p* < 0.001) seizures, respectively, compared with the vehicle (Figure 11a,a’). Regarding the dSWDs, the peak effectiveness was observed at 120 min, where the doses of 15 mg/kg, 10 mg/kg and 5 mg/kg showed a significant reduction (*p* < 0.001) in duration, with respective reductions of 73.33, 64.48, and 24.26 s compared with the vehicle (Figure 11b,b’).

**Figure 12:**
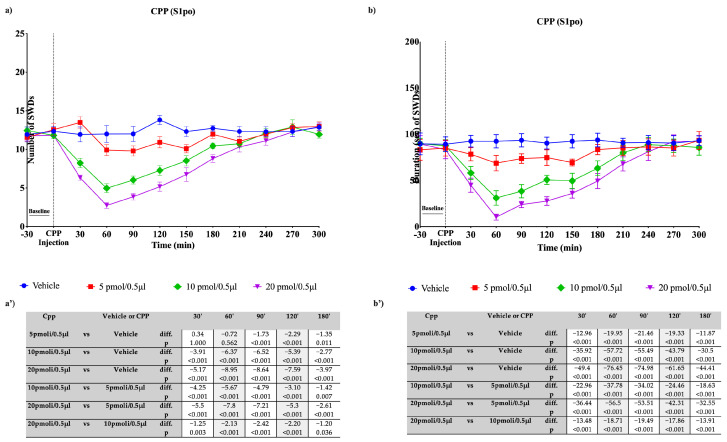
Effects of focal administration into the S1po of different doses of CPP on SWDs in WAG/Rij rats. Plots show the effects of RLZ, at the three doses used, and vehicle on the number (**a**) and duration (**b**) of SDWs in WAG/Rij rats. Tables report the pairwise comparisons between doses on the number (**a’**) and duration (**b’**) of SDWs in WAG/Rij rats, performed at the most significant time points, namely 30′, 60′, 90′, 120′, and 180′. Bonferroni correction was performed. Data are expressed as means ± standard error of the mean (SEM) of 8 animals for each dose. CPP: 3-(2-carboxypiperazine-4-yl)-1-phosphonate; i.p.: intraperitoneal; S1po: primary somatosensory cortex; SWDs: spike-wave discharges.

Likewise, the focal bilateral injection of CPP into the S1po produced a significant (*p* < 0.001) reduction in the SWD parameters. This effect was observed immediately after injection, reaching a peak at 60 min (Figure 12a,b). This antiseizure effect was more prominent at 20 pmol/0.5 µL (−8.95 seizures compared with the vehicle; *p* < 0.001). Pairwise comparisons are reported in Figure 12a’,b’.

### 3.5. Effects of Combined Administration of RLZ with Glutamate Receptor Antagonists in WAG/Rij Rats

**Figure 13:**
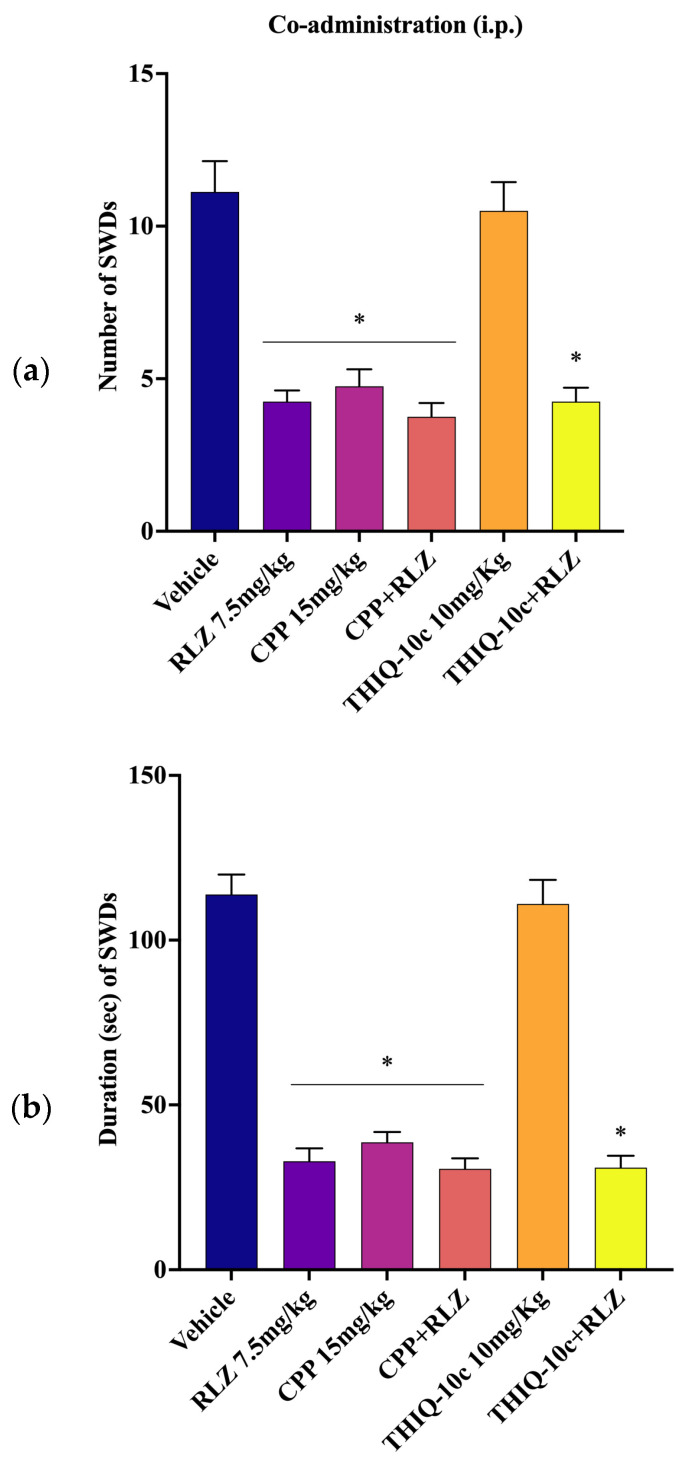
Intraperitoneal effects of RLZ, CPP, THIQ-10c, and their co-treatment on number (**a**) and duration (**b**) of SWDs in WAG/Rij rats. Data are expressed as means ± standard error of the mean (SEM) of 8 animals for each dose. * *p* < 0.05 vs. vehicle (control) group. CPP: 3-(2-carboxypiperazine-4-yl)-1-phosphonate; i.p.: intraperitoneal; SWDs: spike-wave discharges; RLZ: Riluzole; THIQ: N-Acetyl-1-(4-chlorophenyl)-6,7-dimethoxy-1,2,3,4-tetrahydroisoquinoline.

We previously demonstrated that THIQ-10c (0.3, 1, 3, or 10 mg/kg, i.p.), a non-competitive AMPA receptor antagonist, was unable to change the occurrence of SWDs in WAG/Rij rats. At odds, THIQ-10c administration (1, 5, 10, or 20 µg/mL) into S1po led to a dose-dependent reduction in the SWD parameters without behavioral alterations [29,30]. Not surprisingly, RLZ at 7.5 mg/kg and CPP at 15 mg/kg were able to significantly reduce the number and duration of SWDs in WAG/Rij rats. However, the co-administration of CPP (at the highest protective dose, 15 mg/kg) with RLZ (7.5 mg/kg i.p.) induced a slight not-significant effect on SWD parameters compared with RLZ alone (Figure 13a,b). Similarly, the co-administration of AMPA receptor antagonist THIQ-10c (10 mg/kg i.p.) with RLZ (7.5 mg/kg i.p.) did not significantly change the SWD parameters compared with RLZ alone (Figure 13a,b).

**Figure 14:**
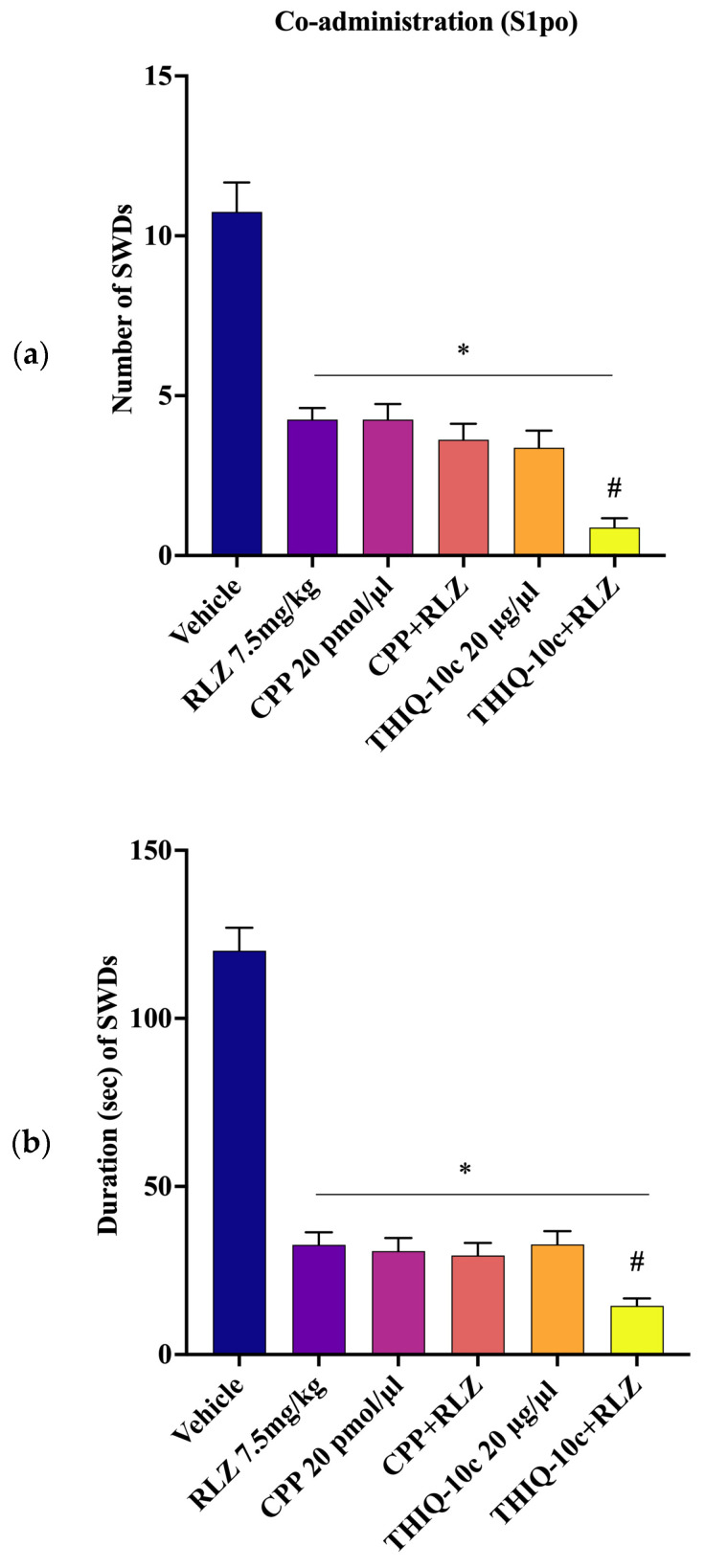
Focal effects of RLZ, CPP, THIQ-10c, and their co-treatment on number (**a**) and duration (**b**) of SWDs in WAG/Rij rats. Data are expressed as means ± standard error of the mean (SEM) of 8 animals for each dose. * *p* < 0.05 vs. vehicle (control) group. # *p* < 0.05 vs. RLZ alone. CPP: 3-(2-carboxypiperazine-4-yl)-1-phosphonate; i.p.: intraperitoneal; S1po: primary somatosensory cortex; SWDs: spike-wave discharges; RLZ: Riluzole; THIQ = N-Acetyl-1-(4-chlorophenyl)-6,7-dimethoxy-1,2,3,4-tetrahydroisoquinoline.

Similarly, the co-administration of CPP, focally administered at 20 pmol/µL into S1po, with RLZ (7.5 mg/kg i.p.), resulted in a not significant reduction in the number and duration of SWDs compared with the administration of RLZ alone. Interestingly, the co-administration of THIQ-10c (at the highest protective dose, 20 µg/µL) into S1po, with RLZ (7.5 mg/kg i.p.) exerted additive effects (*p* < 0.05) in reducing both the number and duration of SWDs compared with RLZ alone (Figure 14a,b).

## 4. Discussion

Glutamate neurotransmitter dysfunction is linked to neurotoxic effects, causing neuronal death and different neuropsychiatric disorders, including epilepsy [41]. Excitatory amino acid antagonists have shown anti-convulsant activity in several animal models of seizures, offering new therapeutic perspectives to treat epilepsy [12,42,43]. The present study shows that RLZ was effective against limbic seizures, induced by glutamatergic agonists in Sprague–Dawley rats, and against established absence seizure in the WAG/Rij rat.

### 4.1. Effects of RLZ against Limbic Seizure in Sprague–Dawley Rats

RLZ has been shown to have anti-convulsant effects by unclear mechanisms of action [19,20,22].

To date, it is known that RLZ blocks the glutamatergic system through several mechanisms. However, limited studies focus on whether or not RLZ blocks selectively the ionotropic glutamate receptors [44]. Because of this, we studied the potential antiseizure effects of RLZ against limbic seizure induced by ionotropic glutamatergic receptors agonists. We found that RLZ, at 1, 5, and 7.5 mg/kg, reduced the seizures severity score in NMDA-treated SD rats. Based on this, we can speculate that these effects of RLZ can be mainly mediated by NMDA receptors. Our hypothesis is supported by a previous study, performed on neuronal cultures and brain slices, in which it has been demonstrated that RLZ can act as an allosteric modulator of the NMDA receptors [45]. In fact, RLZ effects seem to be due to its binding with the glycine/NMDA receptor complex [46,47,48]. Moreover, in this latter study, it has also been observed that RLZ is more effective against NMDA-elicited currents than against KA-elicited currents [45]. Similarly, the efficacy of RLZ on seizure induced by amygdala kindling has been ascribed to its antagonism with the NMDA receptor [22]. Herein, we also found a protective effect of RLZ on AMPA and KA-induced limbic seizure only at the higher doses used, supporting the hypothesis that RLZ can preferentially interact with NMDA receptors. However, we cannot totally exclude that RLZ can also interact with AMPA and KA receptors [17,49,50]. Studies have previously shown how compounds antagonizing the NMDA receptor, including CPP, counteract sound-induced tonic-clonic seizures in GEPRs, a validated model of reflex epilepsy. Interestingly, tolerance does not occur for the antiseizure effects of these drugs [51,52,53]. Similarly, it has been found that RLZ protects against NMDA and AMPA-induced seizure in a genetic model of reflex epilepsy, the DBA/2 mouse [18]. Overall, despite the multi targets effects of RLZ [54,55], its antiseizure properties against limbic seizure, can be mainly due to its activity on NMDA receptors rather than other inotropic glutamate receptors [6,56].

### 4.2. Effects of RLZ against Absence Seizure in WAG/Rij Rats

In the WAG/Rij rats, systemic administration of RLZ exerts significant anti-absence effects decreasing the SWD parameters without inducing a sedative effect in accordance with a previous study [38]. To date, several studies have documented the involvement of NMDA and AMPA receptors in the development and maintenance of absence seizure in WAG/Rij rats [23]. In fact, different studies reported that competitive and non-competitive antagonists of NMDA receptors significantly reduce the SWD parameters in WAG/Rij rats [57,58]. On the contrary, AMPA receptor antagonists only have effects on established absence seizures when focally administered in WAG/Rij rats [29,59,60,61]. In fact, in a previous study, we demonstrated that an early long-term treatment with perampanel (3 mg/kg/day orally for 17 weeks starting from P30), a selective non-competitive AMPA receptor antagonist, reduces the epilepsy onset and its associated depressive comorbidity in WAG/Rij rats, showing antiepileptogenic properties. At odds, both a subchronic and an acute perampanel treatment were not able to modify established absence seizures and depressive-like comorbidity in this model. These data indicate that AMPA receptors are implicated in the mechanisms of epileptogenesis in the WAG/Rij rat model and that these mechanisms differ from those responsible for seizure generation and spread when epilepsy is established [61]. Interestingly, in a retrospective study, it has been demonstrated that perampanel was able to reduce the seizure burden in drug-resistant childhood absence epilepsy. According to the authors, randomized, controlled trials are needed to confirm the effectiveness of perampanel in childhood absence epilepsy [62]. Several explanations can justify this contradiction, and thus, further studies are needed. To date, all antiseizure medications affect SWDs activity in the same way in WAG/Rij rats and childhood, with only the exception of lamotrigine [63], confirming the predictive validity of this model. In agreement with our results, the efficacy of RLZ, systematically injected against absence seizure, might be attributed to its ability to antagonize the NMDA receptors, whereas its effect seems to be independent of the blockade of AMPA receptors. Based on the multi targets profile of this drug [6,44], we cannot exclude that other mechanisms of action linked to glutamatergic neurotransmission are responsible to its anti-absence effect of RLZ. We also found that focal RLZ administration, depending on the brain areas of microinjections, led to different effects on the SWD parameters in WAG/Rij rats. A significant decrease in the incidence of SWDs was found only when RLZ was administered into the S1po, while RLZ administration in VPM induced a clinically meaningful reduction at the higher doses on the SWDs parameters. Despite statistical significance, no clinically meaningful reduction was found when RLZ was administered in the NRT. Several explanations may justify this contradiction and therefore, further experiments are warranted. Overall, as previously suggested, this variability can be explained by the interplay between the mechanisms of action possessed by RLZ. In fact, data regarding NMDA agonists and antagonists in absence epilepsy remains controversial [57,64,65]. The abnormal excitation of S1po has been identified as a leading cause of seizure onset in WAG/Rij rats; from here, they quickly spread over the cortex and to thalamic structures [23,66,67].

An increase in synaptic excitability, mediated by NMDA receptors situated in deep neocortical layers, has been reported in the WAG/Rij rat. This S1po hyperexcitability was linked both to an altered expression of NMDA receptor subunits [68,69], and subunits of AMPA receptor in WAG/Rij rats [29,69,70]. Not surprisingly, focal and i.p. administration of CPP, a competitive NMDA receptor antagonist, caused a significant reduction in SWD parameters [23,71]. Accordingly, it can be supposed that RLZ reduces absence seizures by decreasing the glutamatergic activity through the antagonism on NMDA receptors. However, the reduction in SWDs with RLZ administration in the S1po might be associated with an inhibitory effect on glutamatergic neurotransmission mediated by both the glycine/NMDA and AMPA receptor complex. In fact, it is well-shown that the blockade of NMDA receptors alone is not sufficient to completely eliminate epileptic discharges and AMPA receptors play a key role in modulating excitatory synaptic transmission in epilepsy [72]. AMPA antagonists administered systemically do not seem to be very effective drugs against absence seizures, whereas their administration into the S1po induced a significant reduction in SWDs [23]. In accordance with these pieces of evidence, focal administration of THIQ-10c (AMPA receptors antagonists) into S1po induced a decrease in SWD parameters in WAG/Rij rats [29,30].

### 4.3. Effects of Co-Administration of RLZ with NMDA/AMPA Antagonist Receptors in WAG/Rij Rats

To better define the RLZ mechanism of action, we evaluated the effects of the associated administration of RLZ with CPP and THIQ on SWD parameters in WAG/Rij rats. Both i.p. and focal S1po co-administration of competitive NMDA receptor antagonist, CPP with RLZ, were devoid of any significant effect upon the SWD parameters in comparison with RLZ alone. Accordingly, we can also assume that the lack of potentiation of the antiseizure effect of RLZ, in combination with CPP, can be correlated to the interaction on the same site of the NMDA receptor in this animal model. However, regarding the co-administration of RLZ with non-competitive AMPA receptor antagonist THIQ-10c, we found that only the focal S1po co-administration potentiated the protective activity of RLZ, showing additive effects. Interestingly, the observation that the co-administration of THIQ-10c increases the activity profile of RLZ showed that the modulation of AMPA receptors in the presence of RLZ might be therapeutically useful in the treatment of absence epilepsy. This result can be due to the antagonism of THIQ-10c on the high expression of the GluR1 subunit of AMPA receptors in S1po [70]. It is well-known that the inactivation of AMPA receptors in the S1po reduces seizure activities in the absence epileptic rats [29]. Therefore, RLZ potently attenuates the occurrence of SWDs in WAG/Rij rats probably by acting mainly on NMDA receptors. In fact, the RLZ co-treatment with CPP into the S1po did not induce a marked efficacy in reducing SWD parameters more than RLZ alone. On the contrary, the co-administration of RLZ with THIQ-10c into the S1po led to a significant reduction in SWD parameters compared with RLZ alone, supporting an additive effect.

## 5. Conclusions

In our current study, we showed that RLZ exhibits an antiseizure effect in both models of epilepsy, acting mainly on the NMDA glutamate receptor. Further studies are needed to explain the anti-convulsant effects of RLZ in other different models of epilepsy. Similarly, these studies should also investigate the precise mechanisms linked to the RLZ antiseizure properties. Moreover, it should also be investigated the effectiveness of RLZ in counteracting the neuropsychiatric comorbidities, such as cognitive impairment and depression related to epilepsy, which, as widely reported in the literature, are sometimes more harmful than seizures themselves.

## Data Availability

Not applicable.

## Supplementary Materials

The following supporting information can be downloaded at: https://www.mdpi.com/article/10.3390/pharmaceutics15072006/s1. Details of the predictive model and pairwise comparisons are given below for each of the figures.

## References

[1] Andrews J.A., Jackson C.E., Heiman-Patterson T.D., Bettica P., Brooks B.R., Pioro E.P. (2020). Real-world evidence of riluzole effectiveness in treating amyotrophic lateral sclerosis. Amyotroph. Lateral Scler. Front. Degener..

[2] Nikbakht A., Kargar-soleimanabad S., Siahposht-Khachaki A., Farzin D. (2022). The effect of Riluzole on neurological outcomes, blood-brain barrier, brain water and neuroinflammation in traumatic brain injury. Brain Disord..

[3] Matthews D.C., Mao X., Dowd K., Tsakanikas D., Jiang C.S., Meuser C., Andrews R.D., Lukic A.S., Lee J., Hampilos N. (2021). Riluzole, a glutamate modulator, slows cerebral glucose metabolism decline in patients with Alzheimer’s disease. Brain.

[4] Liu J., Wang L.N. (2018). The efficacy and safety of riluzole for neurodegenerative movement disorders: A systematic review with meta-analysis. Drug Deliv..

[5] Lazarevic V., Yang Y., Ivanova D., Fejtova A., Svenningsson P. (2018). Riluzole attenuates the efficacy of glutamatergic transmission by interfering with the size of the readily releasable neurotransmitter pool. Neuropharmacology.

[6] Bellingham M.C. (2011). A Review of the Neural Mechanisms of Action and Clinical Efficiency of Riluzole in Treating Amyotrophic Lateral Sclerosis: What have we Learned in the Last Decade?. CNS Neurosci. Ther..

[7] Kyllo T., Singh V., Shim H., Latika S., Nguyen H.M., Chen Y.J., Terry E., Wulff H., Erickson J.D. (2023). Riluzole and novel naphthalenyl substituted aminothiazole derivatives prevent acute neural excitotoxic injury in a rat model of temporal lobe epilepsy. Neuropharmacology.

[8] He Y., Benz A., Fu T., Wang M., Covey D.F., Zorumski C.F., Mennerick S. (2002). Neuroprotective agent riluzole potentiates postsynaptic GABAA receptor function. Neuropharmacology.

[9] Jahn K., Schlesinger F., Jin L.J., Dengler R., Bufler J., Krampfl K. (2008). Molecular mechanisms of interaction between the neuroprotective substance riluzole and GABAA-receptors. Naunyn-Schmiedeberg’s Arch. Pharmacol..

[10] Devinsky O., Vezzani A., O’Brien T.J., Jette N., Scheffer I.E., De Curtis M., Perucca P. (2018). Epilepsy.

[11] Hanada T. (2020). Ionotropic glutamate receptors in epilepsy: A review focusing on ampa and nmda receptors. Biomolecules.

[12] Leo A., Giovannini G., Russo E., Meletti S. (2018). The role of AMPA receptors and their antagonists in status epilepticus. Epilepsia.

[13] Rusina E., Bernard C., Williamson A. (2021). The kainic acid models of temporal lobe epilepsy. eNeuro.

[14] Siniscalchi A., Bonci A., Mercuri N.B., Bernardi G. (1997). Effects of riluzole on rat cortical neurones: An in vitro electrophysiological study. Br. J. Pharmacol..

[15] Spadoni F., Hainsworth A.H., Mercuri N.B., Caputi L., Martella G., Lavaroni F., Bernardi G., Stefani A. (2002). Lamotrigine derivatives and riluzole inhibit INa,P in cortical neurons. Neuroreport.

[16] Diao L., Hellier J.L., Uskert-Newsom J., Williams P.A., Staley K.J., Yee A.S. (2013). Diphenytoin, riluzole and lidocaine: Three sodium channel blockers, with different mechanisms of action, decrease hippocampal epileptiform activity. Neuropharmacology.

[17] Zona C., Cavalcanti S., De Sarro G., Siniscalchi A., Marchetti C., Gaetti C., Costa N., Mercuri N., Bernardi G. (2002). Kainate-induced currents in rat cortical neurons in culture are modulated by riluzole. Synapse.

[18] De Sarro G., Siniscalchi A., Ferreri G., Gallelli L., De Sarro A. (2000). NMDA and AMPA/kainate receptors are involved in the anticonvulsant activity of riluzole in DBA/2 mice. Eur. J. Pharmacol..

[19] Zgrajka W., Nieoczym D., Czuczwar M., Kiś J., Brzana W., Wlaź P., Turski W.A. (2010). Evidences for pharmacokinetic interaction of riluzole and topiramate with pilocarpine in pilocarpine-induced seizures in rats. Epilepsy Res..

[20] Kim J.E., Kim D.S., Kwak S.E., Choi H.C., Song H.K., Choi S.Y., Kwon O.S., Kim Y.I., Kang T.C. (2007). Anti-glutamatergic effect of riluzole: Comparison with valproic acid. Neuroscience.

[21] Borowicz K.K., Sekowski A., Drelewska E., Czuczwar S.J. (2004). Riluzole enhances the antiseizure action of conventional antiepileptic drugs against pentetrazole-induced convulsions in mice. Pol. J. Pharmacol..

[22] Yoshida M., Noguchi E., Tsuru N., Ohkoshi N. (2001). Effect of riluzole on the acquisition and expression of amygdala kindling. Epilepsy Res..

[23] Russo E., Citraro R., Constanti A., Leo A., Lüttjohann A., van Luijtelaar G., De Sarro G. (2016). Upholding WAG/Rij rats as a model of absence epileptogenesis: Hidden mechanisms and a new theory on seizure development. Neurosci. Biobehav. Rev..

[24] Leo A., Citraro R., Tallarico M., Iannone M., Fedosova E., Nesci V., De Sarro G., Sarkisova K., Russo E. (2019). Cognitive impairment in the WAG/Rij rat absence model is secondary to absence seizures and depressive-like behavior. Prog. Neuro-Psychopharmacol. Biol. Psychiatry.

[25] Racine R.J. (1972). Modification of seizure activity by electrical stimulation: II. Motor seizure. Electroencephalogr. Clin. Neurophysiol..

[26] Dong X., Fan J., Lin D., Wang X., Kuang H., Gong L., Chen C., Jiang J., Xia N., He D. (2022). Captopril alleviates epilepsy and cognitive impairment by attenuation of C3-mediated inflammation and synaptic phagocytosis. J. Neuroinflamm..

[27] Leo A., Nesci V., Tallarico M., Amodio N., Gallo Cantafio E.M., De Sarro G., Constanti A., Russo E., Citraro R. (2020). IL-6 Receptor Blockade by Tocilizumab Has Anti-absence and Anti-epileptogenic Effects in the WAG/Rij Rat Model of Absence Epilepsy. Neurotherapeutics.

[28] De Sarro G., Ibbadu G.F., Marra R., Rotiroti D., Loiacono A., Donato Di Paola E., Russo E. (2004). Seizure susceptibility to various convulsant stimuli in dystrophin-deficient mdx mice. Neurosci. Res..

[29] Citraro R., Russo E., Gratteri S., Di Paola E.D., Ibbadu G.F., Curinga C., Gitto R., Chimirri A., Donato G., De Sarro G. (2006). Effects of non-competitive AMPA receptor antagonists injected into some brain areas of WAG/Rij rats, an animal model of generalized absence epilepsy. Neuropharmacology.

[30] Russo E., Citraro R., De Fazio S., Marra R., Gitto R., Chimirri A., De Sarro G., Paola E.D. (2008). Di Enhancement of anti-absence effects of ethosuximide by low doses of a noncompetitive α-amino-3-hydroxy-5-methyl-4-isoxazolepropionic acid (AMPA) receptor antagonist in a genetic animal model of absence epilepsy. Epilepsy Behav..

[31] Tallarico M., Leo A., Guarnieri L., Zito M.C., De Caro C., Nicoletti F., Russo E., Constanti A., De Sarro G., Citraro R. (2022). N-acetylcysteine aggravates seizures while improving depressive-like and cognitive impairment comorbidities in the WAG/Rij rat model of absence epilepsy. Mol. Neurobiol..

[32] Palkovits M. (1983). The rat brain in stereotaxic coordinates. Neuropeptides.

[33] Leo A., Citraro R., Amodio N., De Sarro C., Gallo Cantafio M.E., Constanti A., De Sarro G., Russo E. (2017). Fingolimod Exerts only Temporary Antiepileptogenic Effects but Longer-Lasting Positive Effects on Behavior in the WAG/Rij Rat Absence Epilepsy Model. Neurotherapeutics.

[34] Van Luijtelaar E.L.J.M., Coenen A.M.L. (1986). Two types of electrocortical paroxysms in an inbred strain of rats. Neurosci. Lett..

[35] Andersen P.K. (2003). *Regression Modeling Strategies with: Applications to Linear Models, Logistic Regression and Survival Analysis*; Frank, E., Harrell, J., Eds.; Springer: New York, NY, USA, 2001; p. 568. ISBN 0-387-95232-2. Stat. Med..

[36] Chiu K.M., Wu C.C., Wang M.J., Lee M.Y., Wang S.J. (2015). Protective effects of bupivacaine against kainic acid-induced seizure and neuronal cell death in the rat hippocampus. Biol. Pharm. Bull..

[37] Russo E., Constanti A., Ferreri G., Citraro R., De Sarro G. (2004). Nifedipine affects the anticonvulsant activity of topiramate in various animal models of epilepsy. Neuropharmacology.

[38] Romettino S., Lazdunski M., Gottesmann C. (1991). Anticonvulsant and sleep-waking influences of riluzole in a rat model of absence epilepsy. Eur. J. Pharmacol..

[39] De Sarro G.B., De Sarro A. (1993). Anticonvulsant properties of non-competitive antagonists of the N-methyl-D-aspartate receptor in genetically epilepsy-prone rats: Comparison with CPPene. Neuropharmacology.

[40] De Sarro G., De Sarro A. (1992). Anticonvulsant activity of competitive antagonists of NMDA receptor in genetically epilepsy-prone rats. Eur. J. Pharmacol..

[41] Green J.L., dos Santos W.F., Fontana A.C.K. (2021). Role of glutamate excitotoxicity and glutamate transporter EAAT2 in epilepsy: Opportunities for novel therapeutics development. Biochem. Pharmacol..

[42] Andersen J.V., Schousboe A. (2022). Glial Glutamine Homeostasis in Health and Disease. Neurochem. Res..

[43] Löscher W., Klein P. (2021). The Pharmacology and Clinical Efficacy of Antiseizure Medications: From Bromide Salts to Cenobamate and Beyond. CNS Drugs.

[44] Bissaro M., Moro S. (2019). Rethinking to riluzole mechanism of action: The molecular link among protein kinase CK1δ activity, TDP-43 phosphorylation, and amyotrophic lateral sclerosis pharmacological treatment. Neural Regen. Res..

[45] Debono M.W., Le Guern J., Canton T., Doble A., Pradier L. (1993). Inhibition by riluzole of electrophysiological responses mediated by rat kainate and NMDA receptors expressed in Xenopus oocytes. Eur. J. Pharmacol..

[46] Doble A. (1996). The pharmacology and mechanism of action of riluzole. Neurology.

[47] Kretschmer B.D., Kratzer U., Schmidt W.J. (1998). Riluzole, a glutamate release inhibitor, and motor behavior. Naunyn-Schmiedeberg’s Arch. Pharmacol..

[48] Lamanauskas N., Nistri A. (2008). Riluzole blocks persistent Na+ and Ca2+ currents and modulates release of glutamate via presynaptic NMDA receptors on neonatal rat hypoglossal motoneurons in vitro. Eur. J. Neurosci..

[49] Jin L.J., Schlesinger F., Song Y.P., Dengler R., Krampfl K. (2010). The interaction of the neuroprotective compounds riluzole and phenobarbital with AMPA-type glutamate receptors: A patch-clamp study. Pharmacology.

[50] Centonze D., Calabresi P., Pisani A., Marinelli S., Marfia G.A., Bernardi G. (1998). Electrophysiology of the neuroprotective agent riluzole on striatal spiny neurons. Neuropharmacology.

[51] Smith S.E., Chapman A.G. (1993). Acute and chronic anticonvulsant effects of d(-)CPPene in genetically epilepsy-prone rats. Epilepsy Res..

[52] Faingold C.L., Randall M.E., Naritoku D.K., Boersma Anderson C.A. (1993). Noncompetitive and competitive NMDA antagonists exert anticonvulsant effects by actions on different sites within the neuronal network for audiogenic seizures. Exp. Neurol..

[53] De Sarro C., Tallarico M., Pisano M., Gallelli L., Citraro R., De Sarro G., Leo A. (2022). Liraglutide chronic treatment prevents development of tolerance to antiseizure effects of diazepam in genetically epilepsy prone rats. Eur. J. Pharmacol..

[54] Wang S.J., Wang K.Y., Wang W.C. (2004). Mechanisms underlying the riluzole inhibition of glutamate release from rat cerebral cortex nerve terminals (synaptosomes). Neuroscience.

[55] Martin D., Thompson M.A., Nadler J.V. (1993). The neuroprotective agent riluzole inhibits release of glutamate and aspartate from slices of hippocampal area CA1. Eur. J. Pharmacol..

[56] Dorst J., Ludolph A.C., Huebers A. (2018). Disease-modifying and symptomatic treatment of amyotrophic lateral sclerosis. Ther. Adv. Neurol. Disord..

[57] Peeters B.W.M.M., van Rijn C.M., Vossen J.M.H., Coenen A.M.L. (1990). Involvement of NMDA receptors in non-convulsive epilepsy in WAG/Rij rats. Life Sci..

[58] Peeters B.W.M.M., Ramakers G.M.J., Vossen J.M.H., Coenen A.M.L. (1994). The WAG/Rij Rat Model for Nonconvulsive Absence Epilepsy: Involvement of NonNMDA Receptors. Brain Res. Bull..

[59] Kaminski R.M., Van Rijn C.M., Turski W.A., Czuczwar S.J., Van Luijtelaar G. (2001). AMPA and GABA(B) receptor antagonists and their interaction in rats with a genetic form of absence epilepsy. Eur. J. Pharmacol..

[60] Jakus R., Graf M., Ando R.D., Balogh B., Gacsalyi I., Levay G., Kantor S., Bagdy G. (2004). Effect of two noncompetitive AMPA receptor antagonists GYKI 52466 and GYKI 53405 on vigilance, behavior and spike-wave discharges in a genetic rat model of absence epilepsy. Brain Res..

[61] Citraro R., Leo A., Franco V., Marchiselli R., Perucca E., De Sarro G., Russo E. (2017). Perampanel effects in the WAG/Rij rat model of epileptogenesis, absence epilepsy, and comorbid depressive-like behavior. Epilepsia.

[62] Operto F.F., Orsini A., Sica G., Scuoppo C., Padovano C., Vivenzio V., de Simone V., Rinaldi R., Belfiore G., Mazza R. (2022). Perampanel and childhood absence epilepsy: A real life experience. Front. Neurol..

[63] Coenen A.M.L., Van Luijtelaar E.L.J.M. (2003). Genetic Animal Models for Absence Epilepsy: A Review of the WAG/Rij Strain of Rats. Behav. Genet..

[64] Peeters B.W., van Rijn C.M., Van Luijtelaar E.L., Coenen A.M. (1989). Antiepileptic and behavioural actions of MK-801 in an animal model of spontaneous absence epilepsy. Epilepsy Res..

[65] Peeters B.W.M.M., Ramakers G.M.J., Ellenbroek B.A., Vossen J.M.H., Coenen A.M.L. (1994). Interactions between NMDA and NonNMDA Receptors in Nonconvulsive Epilepsy in the WAG/Rij Inbred Strain. Brain Res. Bull..

[66] Meeren H., Van Luijtelaar G., Lopes Da Silva F., Coenen A. (2005). Evolving concepts on the pathophysiology of absence seizures: The cortical focus theory. Arch. Neurol..

[67] Touret M., Parrot S., Denoroy L., Belin M.F., Didier-Bazes M. (2007). Glutamatergic alterations in the cortex of genetic absence epilepsy rats. BMC Neurosci..

[68] Karimzadeh F., Soleimani M., Mehdizadeh M., Jafarian M., Mohamadpour M., Kazemi H., Joghataei M.T., Gorji A. (2013). Diminution of the NMDA receptor NR2B subunit in cortical and subcortical areas of WAG/Rij rats. Synapse.

[69] Van de Bovenkamp-Janssen M.C., van der Kloet J.C., van Luijtelaar G., Roubos E.W. (2006). NMDA-NR1 and AMPA-GluR4 receptor subunit immunoreactivities in the absence epileptic WAG/Rij rat. Epilepsy Res..

[70] Zavvari F., Mousavi S.M.M., Ejlali M., Barfi S., Karimzadeh F. (2020). Glutamate signaling pathway in absence epilepsy: Possible role of ionotropic ampa glutamate receptor type 1 subunit. Iran. J. Pharm. Res..

[71] D’Antuono M., Inaba Y., Biagini G., D’Arcangelo G., Tancredi V., Avoli M. (2006). Synaptic hyperexcitability of deep layer neocortical cells in a genetic model of absence seizures. Genes Brain Behav..

[72] Rogawski M.A. (2011). Revisiting AMPA receptors as an antiepileptic drug target. Epilepsy Curr..

