# Supplementary material for: An In Vivo Electroencephalographic Analysis of the Effect of Riluzole against Limbic and Absence Seizure and Comparison with Glutamate Antagonists
Source: Pharmaceutics. 2023 Jul 22;15(7):2006. doi: 10.3390/pharmaceutics15072006 (PMC10386681; doi:10.3390/pharmaceutics15072006)
Supplement: Supplementary file 1 [file pharmaceutics-15-02006-s001.zip › pharmaceutics-2242094-supplementary.pdf]

Supplementary materials

Details of the predictive model and pairwise comparisons are given below for each of the figures. The standardized normal probability plot is also shown for Figures 7-12.

Summary

Figure 4. Kainate 2
Figure 5. NMDA 4
Figure 6. AMPA 6
Figure 7. dSWDs........ 9
Figure 7. nSWDs........ 14
Figure 8. dSWDs........ 19
Figure 8. nSWDs........ 23
Figure 9. dSWDs........ 27
Figure 9. nSWDs........ 31
Figure 10. dSWDs........ 35
Figure 10. nSWDs........ 39
Figure 11. dSWDs........ 43
Figure 11. nSWDs........ 47
Figure 12. dSWDs........ 51
Figure 12. nSWDs........ 55

Figure 4. Kainate

	Odds ratio	Std. err.	z	P>|z|	[95% conf.	interval]
dose						
1	.6828332	.7167615	-0.36	0.716	.0872607	5.34331
2	.0005935	.0009226	-4.78	0.000	.0000282	.0124943
3	1.49e-06	2.89e-06	-6.91	0.000	3.32e-08	.0000668
4	1.16e-06	2.29e-06	-6.95	0.000	2.46e-08	.000055
time						
60	6.8864	7.75482	1.71	0.087	.7576153	62.59445
120	16.8396	22.70872	2.09	0.036	1.19801	236.7027
180	16.97877	22.90766	2.10	0.036	1.206341	238.9695
dose#time						
1 60	1.36716	2.121306	0.20	0.840	.0653255	28.61251
1 120	.5590876	.9569951	-0.34	0.734	.0195196	16.0136
1 180	.557381	.9537965	-0.34	0.733	.019479	15.94916
2 60	5.425655	8.776192	1.05	0.296	.2278258	129.2115
2 120	1513.276	3268.6	3.39	0.001	21.94649	104344.8
2 180	492.7674	1002.14	3.05	0.002	9.152816	26529.51
3 60	1.906933	3.102177	0.40	0.692	.078634	46.24453
3 120	23.54179	44.83849	1.66	0.097	.5631344	984.1625
3 180	51.49656	100.5479	2.02	0.044	1.121484	2364.63
4 60	2.396902	3.956353	0.53	0.596	.0943295	60.90503
4 120	28.93713	56.06556	1.74	0.082	.6490179	1290.192
4 180	78.47254	154.2785	2.22	0.026	1.664329	3699.954

dose#time	Odds ratio	Std. err.	z	Adjusted P>|z|
(1 30) vs (0 30)	0,682833	0,716762	-0,36	1.000
(1 60) vs (0 60)	0,933543	1,191829	-0,05	1.000
(1 120) vs (0 120)	0,381764	0,560812	-0,66	1.000
(1 180) vs (0 180)	0,380598	0,558851	-0,66	1.000
(2 30) vs (0 30)	0,000594	0,000923	-4,78	<0.001

(2 30) vs (1 30)	0,000869	0,001339	-4,58	<0.001
(2 60) vs (0 60)	0,00322	0,004604	-4,01	<0.001
(2 60) vs (1 60)	0,003449	0,004859	-4,02	<0.001
(2 120) vs (0 120)	0,898066	1,460203	-0,07	1.000
(2 120) vs (1 120)	2,352415	3,406125	0,59	1.000
(2 180) vs (0 180)	0,292437	0,434008	-0,83	1.000
(2 180) vs (1 180)	0,768361	0,981941	-0,21	1.000
(3 30) vs (0 30)	1,49E-06	2,89E-06	-6,91	<0.001
(3 30) vs (1 30)	2,18E-06	4,2E-06	-6,77	<0.001
(3 30) vs (2 30)	0,002511	0,003701	-4,06	<0.001
(3 60) vs (0 60)	2,84E-06	5,5E-06	-6,59	<0.001
(3 60) vs (1 60)	3,04E-06	5,8E-06	-6,67	<0.001
(3 60) vs (2 60)	0,000882	0,00129	-4,81	<0.001
(3 120) vs (0 120)	3,51E-05	6,69E-05	-5,38	<0.001
(3 120) vs (1 120)	9,19E-05	0,000157	-5,43	<0.001
(3 120) vs (2 120)	3,91E-05	7,28E-05	-5,45	<0.001
(3 180) vs (0 180)	7,67E-05	0,000144	-5,05	<0.001
(3 180) vs (1 180)	0,000202	0,000339	-5,06	<0.001
(3 180) vs (2 180)	0,000262	0,00044	-4,92	<0.001
(4 30) vs (0 30)	1,16E-06	2,29E-06	-6,95	<0.001
(4 30) vs (1 30)	1,71E-06	3,33E-06	-6,8	<0.001
(4 30) vs (2 30)	0,001962	0,002968	-4,12	<0.001
(4 30) vs (3 30)	0,781573	0,9063	-0,21	1.000
(4 60) vs (0 60)	2,79E-06	5,34E-06	-6,68	<0.001
(4 60) vs (1 60)	2,99E-06	5,63E-06	-6,76	<0.001
(4 60) vs (2 60)	0,000867	0,001246	-4,91	<0.001
(4 60) vs (3 60)	0,982391	1,13095	-0,02	1.000
(4 120) vs (0 120)	3,37E-05	6,45E-05	-5,38	<0.001
(4 120) vs (1 120)	8,83E-05	0,000152	-5,44	<0.001
(4 120) vs (2 120)	3,75E-05	7,02E-05	-5,45	<0.001
(4 120) vs (3 120)	0,960696	1,112515	-0,03	1.000
(4 180) vs (0 180)	9,14E-05	0,000168	-5,05	<0.001
(4 180) vs (1 180)	0,00024	0,000395	-5,07	<0.001
(4 180) vs (2 180)	0,000313	0,000511	-4,93	<0.001
(4 180) vs (3 180)	1,190993	1,376949	0,15	1.000

Figure 5. NMDA

	Odds ratio	Std. err.	z	P>|z|	[95% conf. interval]	
dose						
1	.8222685	1.403916	-0.11	0.909	.0289533	23.35228
2	.0008175	.0015924	-3.65	0.000	.000018	.0371947
3	.0012671	.0024263	-3.48	0.000	.0000297	.0540347
4	1.34e-07	3.78e-07	-5.60	0.000	5.22e-10	.0000342
time						
60	37.10697	63.64475	2.11	0.035	1.286769	1070.066
120	14.01962	21.31596	1.74	0.082	.7120953	276.0163
180	26.41636	45.38725	1.91	0.057	.9107139	766.2387
dose#time						
1 60	.8659429	1.889058	-0.07	0.947	.012039	62.28578
1 120	.8892055	1.68616	-0.06	0.951	.0216226	36.56766
1 180	1.14164	2.502849	0.06	0.952	.0155387	83.87696
2 60	.1112184	.2237108	-1.09	0.275	.0021579	5.73232
2 120	.1743823	.3238442	-0.94	0.347	.0045787	6.641453
2 180	.2577595	.5196109	-0.67	0.501	.0049579	13.4008
3 60	.0427238	.0858564	-1.57	0.117	.000832	2.1939
3 120	.0653301	.1217729	-1.46	0.143	.0016924	2.521864
3 180	.0919205	.1846358	-1.19	0.235	.0017933	4.711678
4 60	.9173558	2.107112	-0.04	0.970	.0101713	82.73652
4 120	11.37029	25.61177	1.08	0.280	.1375386	939.9791
4 180	11.73415	28.34725	1.02	0.308	.1030689	1335.906

dose#time	Odds ratio	Std. err.	z	Adjusted P>|z|
(1 30) vs (0 30)	0,822269	1,403916	-0,11	1.000
(1 60) vs (0 60)	0,712038	1,465048	-0,17	1.000
(1 120) vs (0 120)	0,731166	1,287434	-0,18	1.000
(1 180) vs (0 180)	0,938734	1,940545	-0,03	1.000
(2 30) vs (0 30)	0,000818	0,001592	-3,65	<0.001

(2 30) vs (1 30)	0,000994	0,001782	-3,86	<0.001
(2 60) vs (0 60)	9,09E-05	0,000191	-4,44	<0.001
(2 60) vs (1 60)	0,000128	0,000257	-4,46	<0.001
(2 120) vs (0 120)	0,000143	0,00028	-4,51	<0.001
(2 120) vs (1 120)	0,000195	0,000367	-4,54	<0.001
(2 180) vs (0 180)	0,000211	0,000435	-4,1	<0.001
(2 180) vs (1 180)	0,000225	0,000446	-4,23	<0.001
(3 30) vs (0 30)	0,001267	0,002426	-3,48	<0.001
(3 30) vs (1 30)	0,001541	0,002703	-3,69	<0.001
(3 30) vs (2 30)	1,550013	2,425151	0,28	1.000
(3 60) vs (0 60)	5,41E-05	0,000115	-4,62	<0.001
(3 60) vs (1 60)	0,000076	0,000155	-4,66	<0.001
(3 60) vs (2 60)	0,595428	0,901617	-0,34	1.000
(3 120) vs (0 120)	8,28E-05	0,000165	-4,71	<0.001
(3 120) vs (1 120)	0,000113	0,000216	-4,75	<0.001
(3 120) vs (2 120)	0,580693	0,890282	-0,35	1.000
(3 180) vs (0 180)	0,000117	0,000244	-4,33	<0.001
(3 180) vs (1 180)	0,000124	0,00025	-4,47	<0.001
(3 180) vs (2 180)	0,552756	0,838187	-0,39	1.000
(4 30) vs (0 30)	1,34E-07	3,78E-07	-5,6	<0.001
(4 30) vs (1 30)	1,62E-07	4,47E-07	-5,68	<0.001
(4 30) vs (2 30)	0,000163	0,000387	-3,68	<0.001
(4 30) vs (3 30)	0,000105	0,00025	-3,86	<0.001
(4 60) vs (0 60)	1,23E-07	3,2E-07	-6,1	<0.001
(4 60) vs (1 60)	1,72E-07	4,31E-07	-6,23	<0.001
(4 60) vs (2 60)	0,001348	0,002545	-3,5	<0.001
(4 60) vs (3 60)	0,002264	0,004215	-3,27	0,01
(4 120) vs (0 120)	1,52E-06	3,51E-06	-5,81	<0.001
(4 120) vs (1 120)	2,08E-06	4,59E-06	-5,92	<0.001
(4 120) vs (2 120)	0,010656	0,018232	-2,65	0,08
(4 120) vs (3 120)	0,018351	0,031004	-2,37	0,18
(4 180) vs (0 180)	1,57E-06	3,75E-06	-5,59	<0.001
(4 180) vs (1 180)	1,67E-06	3,84E-06	-5,79	<0.001
(4 180) vs (2 180)	0,00744	0,01262	-2,89	0,04
(4 180) vs (3 180)	0,01346	0,022532	-2,57	0,1

Figure 6. AMPA

	Odds ratio	Std. err.	z	P>|z|	[95% conf.	interval]
dose						
1	1.330599	1.217643	0.31	0.755	.2213634	7.998138
2	.999949	.9286379	-0.00	1.000	.1619868	6.172715
3	.0004024	.0005078	-6.19	0.000	.0000339	.0047742
4	.0004216	.0005299	-6.18	0.000	.0000359	.0049506
time						
60	17.85303	22.80063	2.26	0.024	1.46089	218.1757
120	20.70689	26.27898	2.39	0.017	1.721315	249.0975
180	20.70677	26.27879	2.39	0.017	1.721312	249.0951
dose#time						
1 60	.8709128	1.541772	-0.08	0.938	.027108	27.98028
1 120	.6473936	1.146173	-0.25	0.806	.0201449	20.80521
1 180	.1928919	.3038365	-1.04	0.296	.008801	4.227628
2 60	.5114182	.8364494	-0.41	0.682	.0207298	12.61705
2 120	.4408657	.7181633	-0.50	0.615	.0181014	10.73743
2 180	.3727504	.6101773	-0.60	0.547	.0150673	9.221482
3 60	.3001461	.4723985	-0.76	0.444	.0137287	6.561979
3 120	.3571255	.5551683	-0.66	0.508	.0169663	7.517155
3 180	1.74594	2.866957	0.34	0.734	.0698709	43.62769
4 60	.2054091	.3284709	-0.99	0.322	.0089425	4.718254
4 120	.346309	.5448732	-0.67	0.500	.0158565	7.563474
4 180	1.678316	2.715933	0.32	0.749	.0703749	40.02486

dose#time	Odds ratio	Std. err.	z	Adjusted P>|z|
(1 30) vs (0 30)	1,330599	1,217643	0,31	1.000
(1 60) vs (0 60)	1,158836	1,756229	0,1	1.000
(1 120) vs (0 120)	0,861422	1,305499	-0,1	1.000
(1 180) vs (0 180)	0,256662	0,328807	-1,06	1.000
(2 30) vs (0 30)	0,999949	0,928638	0	1.000
(2 30) vs (1 30)	0,751503	0,687706	-0,31	1.000
(2 60) vs (0 60)	0,511392	0,688495	-0,5	1.000
(2 60) vs (1 60)	0,441298	0,590488	-0,61	1.000
(2 120) vs (0 120)	0,440843	0,589995	-0,61	1.000
(2 120) vs (1 120)	0,511763	0,688843	-0,5	1.000
(2 180) vs (0 180)	0,372731	0,502453	-0,73	1.000
(2 180) vs (1 180)	1,452227	1,578484	0,34	1.000
(3 30) vs (0 30)	0,000402	0,000508	-6,19	<0.001
(3 30) vs (1 30)	0,000302	0,000383	-6,4	<0.001
(3 30) vs (2 30)	0,000402	0,000508	-6,19	<0.001
(3 60) vs (0 60)	0,000121	0,000187	-5,84	<0.001
(3 60) vs (1 60)	0,000104	0,00016	-5,96	<0.001
(3 60) vs (2 60)	0,000236	0,000324	-6,1	<0.001
(3 120) vs (0 120)	0,000144	0,000218	-5,83	<0.001
(3 120) vs (1 120)	0,000167	0,000254	-5,71	<0.001
(3 120) vs (2 120)	0,000326	0,000438	-5,97	<0.001
(3 180) vs (0 180)	0,000703	0,001058	-4,82	<0.001
(3 180) vs (1 180)	0,002737	0,003477	-4,65	<0.001
(3 180) vs (2 180)	0,001885	0,002525	-4,68	<0.001
(4 30) vs (0 30)	0,000422	0,00053	-6,18	<0.001
(4 30) vs (1 30)	0,000317	0,000399	-6,39	<0.001
(4 30) vs (2 30)	0,000422	0,00053	-6,18	<0.001
(4 30) vs (3 30)	1,047773	0,852924	0,06	1.000
(4 60) vs (0 60)	8,66E-05	0,000138	-5,89	<0.001
(4 60) vs (1 60)	7,47E-05	0,000118	-6	<0.001
(4 60) vs (2 60)	0,000169	0,00024	-6,12	<0.001
(4 60) vs (3 60)	0,717058	0,710815	-0,34	1.000
(4 120) vs (0 120)	0,000146	0,000224	-5,77	<0.001
(4 120) vs (1 120)	0,00017	0,00026	-5,65	<0.001
(4 120) vs (2 120)	0,000331	0,00045	-5,89	<0.001
(4 120) vs (3 120)	1,016039	0,909064	0,02	1.000

(4 180) vs (0 180)	0,000708	0,001053	-4,87	<0.001
(4 180) vs (1 180)	0,002757	0,003442	-4,72	<0.001
(4 180) vs (2 180)	0,001899	0,002504	-4,75	<0.001
(4 180) vs (3 180)	1,00719	1,008357	0,01	1.000

Figure 7. dSWDs

	Coefficient	Std. err.	z	P>|z|	[95% conf.	interval]
dose						
1	-3.457624	3.791461	-0.91	0.362	-10.88875	3.973502
2	8.037342	3.785055	2.12	0.034	.6187711	15.45591
3	11.626	3.789235	3.07	0.002	4.199235	19.05276
4	5.589016	3.795904	1.47	0.141	-1.850819	13.02885
timeknot1	-.0884164	.0908165	-0.97	0.330	-.2664136	.0895807
timeknot2	.8547379	.8277951	1.03	0.302	-.7677106	2.477186
timeknot3	-2.448322	2.538848	-0.96	0.335	-7.424373	2.527729
timeknot4	2.382087	3.506721	0.68	0.497	-4.490959	9.255134
timeknot5	-.0392662	3.50672	-0.01	0.991	-6.912311	6.833779
dose#c.timeknot1						
1	.296521	.128434	2.31	0.021	.044795	.548247
2	-.0244965	.128434	-0.19	0.849	-.2762225	.2272295
3	-.4398051	.128434	-3.42	0.001	-.6915311	-.1880791
4	-.7148629	.128434	-5.57	0.000	-.9665889	-.4631369
dose#c.timeknot2						
1	-2.957487	1.170679	-2.53	0.012	-5.251976	-.6629988
2	-4.932006	1.170679	-4.21	0.000	-7.226495	-2.637517
3	-3.295722	1.170679	-2.82	0.005	-5.590211	-1.001233
4	-1.27463	1.170679	-1.09	0.276	-3.569119	1.019858
dose#c.timeknot3						
1	9.389731	3.590474	2.62	0.009	2.352532	16.42693
2	21.09261	3.590474	5.87	0.000	14.05541	28.12981
3	17.59704	3.590474	4.90	0.000	10.55984	24.63424
4	11.69714	3.590474	3.26	0.001	4.659945	18.73434
dose#c.timeknot4						
1	-13.47325	4.959252	-2.72	0.007	-23.19321	-3.753297

2	-35.1701	4.959252	-7.09	0.000	-44.89006	-25.45014
3	-29.79965	4.959252	-6.01	0.000	-39.5196	-20.07969
4	-22.35794	4.959252	-4.51	0.000	-32.07789	-12.63798
dose#c.timeknot5						
1	13.24838	4.959251	2.67	0.008	3.528426	22.96833
2	28.76205	4.959251	5.80	0.000	19.04209	38.482
3	17.23898	4.959251	3.48	0.001	7.51903	26.95894
4	11.99959	4.959251	2.42	0.016	2.279639	21.71955
baseline	-.0598782	.0602984	-0.99	0.321	-.1780608	.0583045
_cons	112.0406	6.888952	16.26	0.000	98.53853	125.5427

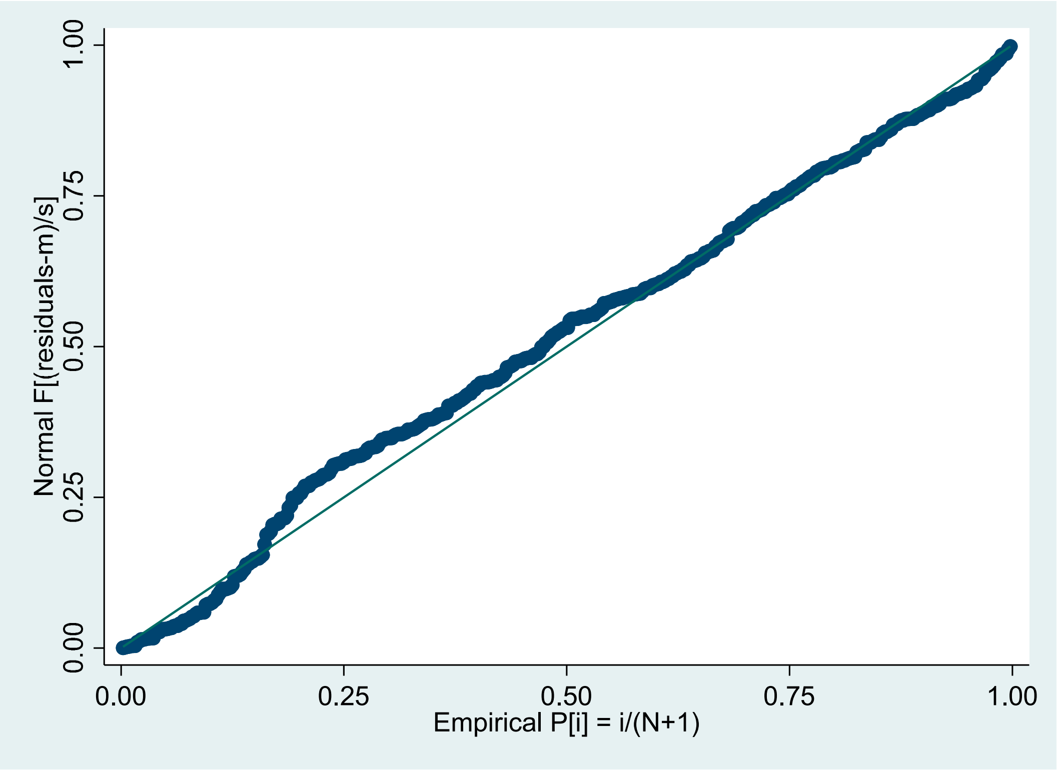

30'

	Contrast	Delta-method std. err.	Bonferroni z	P>|z|
dose				
1 vs 0	4.550761	2.518401	1.81	0.708

	Contrast	std. err.	z	P>|z|
2 vs 0	5.822847	2.508747	2.32	0.203
3 vs 0	-2.556871	2.515049	-1.02	1.000
4 vs 0	-16.23926	2.525085	-6.43	0.000
2 vs 1	1.272086	2.513871	0.51	1.000
3 vs 1	-7.107631	2.508423	-2.83	0.046
4 vs 1	-20.79002	2.50893	-8.29	0.000
3 vs 2	-8.379717	2.511441	-3.34	0.008
4 vs 2	-22.06211	2.519093	-8.76	0.000
4 vs 3	-13.68239	2.510312	-5.45	0.000

60’

	Contrast	Delta-method std. err.	z	Bonferroni P>|z|
dose				
1 vs 0	7.235668	3.008677	2.40	0.162
2 vs 0	-5.26926	3.0006	-1.76	0.791
3 vs 0	-22.67204	3.005871	-7.54	0.000
4 vs 0	-40.36187	3.014274	-13.39	0.000
2 vs 1	-12.50493	3.004886	-4.16	0.000
3 vs 1	-29.90771	3.00033	-9.97	0.000
4 vs 1	-47.59754	3.000754	-15.86	0.000
3 vs 2	-17.40278	3.002853	-5.80	0.000
4 vs 2	-35.09261	3.009256	-11.66	0.000
4 vs 3	-17.68983	3.001909	-5.89	0.000

90’

	Contrast	Delta-method std. err.	z	Bonferroni P>|z|
dose				
1 vs 0	2.090539	2.483316	0.84	1.000
2 vs 0	-27.78881	2.473525	-11.23	0.000
3 vs 0	-49.3727	2.479916	-19.91	0.000

4 vs 0	-65.56401	2.490094	-26.33	0.000
2 vs 1	-29.87935	2.478721	-12.05	0.000
3 vs 1	-51.46324	2.473196	-20.81	0.000
4 vs 1	-67.65455	2.473711	-27.35	0.000
3 vs 2	-21.58389	2.476257	-8.72	0.000
4 vs 2	-37.7752	2.484018	-15.21	0.000
4 vs 3	-16.19131	2.475112	-6.54	0.000

120’

	Contrast	Delta-method std. err.	z	Bonferroni P>|z|
dose				
1 vs 0	-2.123505	3.068395	-0.69	1.000
2 vs 0	-38.97449	3.060477	-12.73	0.000
3 vs 0	-62.19558	3.065644	-20.29	0.000
4 vs 0	-76.59429	3.073884	-24.92	0.000
2 vs 1	-36.85098	3.064678	-12.02	0.000
3 vs 1	-60.07208	3.060211	-19.63	0.000
4 vs 1	-74.47079	3.060627	-24.33	0.000
3 vs 2	-23.2211	3.062685	-7.58	0.000
4 vs 2	-37.6198	3.068964	-12.26	0.000
4 vs 3	-14.39871	3.061759	-4.70	0.000

180’

	Contrast	Delta-method std. err.	z	Bonferroni P>|z|
dose				
1 vs 0	6.218007	3.068395	2.03	0.427
2 vs 0	4.603843	3.060476	1.50	1.000
3 vs 0	-14.75771	3.065644	-4.81	0.000
4 vs 0	-34.75623	3.073883	-11.31	0.000
2 vs 1	-1.614164	3.064678	-0.53	1.000
3 vs 1	-20.97572	3.060211	-6.85	0.000

4 vs 1	-40.97424	3.060627	-13.39	0.000
3 vs 2	-19.36156	3.062685	-6.32	0.000
4 vs 2	-39.36007	3.068963	-12.83	0.000
4 vs 3	-19.99852	3.061759	-6.53	0.000

Figure 7. nSWDs

	Coefficient	Std. err.	z	P>|z|	[95% conf.	interval]
dose						
1	.6133868	.5355905	1.15	0.252	-.4363513	1.663125
2	.6446382	.5353292	1.20	0.229	-.4045878	1.693864
3	-.3284059	.5353946	-0.61	0.540	-1.37776	.7209482
4	-.7431355	.5353946	-1.39	0.165	-1.79249	.3062186
timeknot1	-.0016524	.0128459	-0.13	0.898	-.0268298	.023525
timeknot2	.0439762	.1170904	0.38	0.707	-.1855168	.2734692
timeknot3	-.2018499	.3591164	-0.56	0.574	-.9057051	.5020053
timeknot4	.3633957	.4960206	0.73	0.464	-.6087868	1.335578
timeknot5	-.3075988	.4960205	-0.62	0.535	-1.279781	.6645835
dose#c.timeknot1						
1	-.0420055	.0181668	-2.31	0.021	-.0776118	-.0063992
2	-.0947146	.0181668	-5.21	0.000	-.1303208	-.0591083
3	-.1249524	.0181668	-6.88	0.000	-.1605587	-.0893461
4	-.1423946	.0181668	-7.84	0.000	-.1780008	-.1067883
dose#c.timeknot2						
1	.2538214	.1655908	1.53	0.125	-.0707307	.5783735
2	.3978218	.1655908	2.40	0.016	.0732697	.7223739
3	.6279414	.1655908	3.79	0.000	.3033893	.9524936
4	.6665306	.1655908	4.03	0.000	.3419784	.9910827
dose#c.timeknot3						
1	-.4293999	.5078673	-0.85	0.398	-1.424801	.5660017
2	-.1876883	.5078673	-0.37	0.712	-1.18309	.8077133
3	-.9036875	.5078673	-1.78	0.075	-1.899089	.0917141
4	-.8738586	.5078673	-1.72	0.085	-1.86926	.121543
dose#c.timeknot4						
1	-.2017686	.701479	-0.29	0.774	-1.576642	1.173105

```
           2  |  -1.767701    .701479    -2.52   0.012    -3.142575   -.3928274
           3  |  -.5361369    .701479    -0.76   0.445    -1.911011    .8387368
           4  |  -.7293099    .701479    -1.04   0.298    -2.104184    .6455638
              |
dose#c.timeknot5 |
           1  |   .9159831   .7014789     1.31   0.192    -.4588903    2.290856
           2  |   2.978862   .7014789     4.25   0.000     1.603989    4.353735
           3  |   1.241109   .7014789     1.77   0.077    -.1337644    2.615982
           4  |   1.329312   .7014789     1.90   0.058    -.0455615    2.704185
              |
    baseline  |   .0400174   .0669139     0.60   0.550    -.0911314    .1711662
       _cons  |   12.11016   .9257188    13.08   0.000     10.29579    13.92454
--------------------------------------------------------------------------------
```

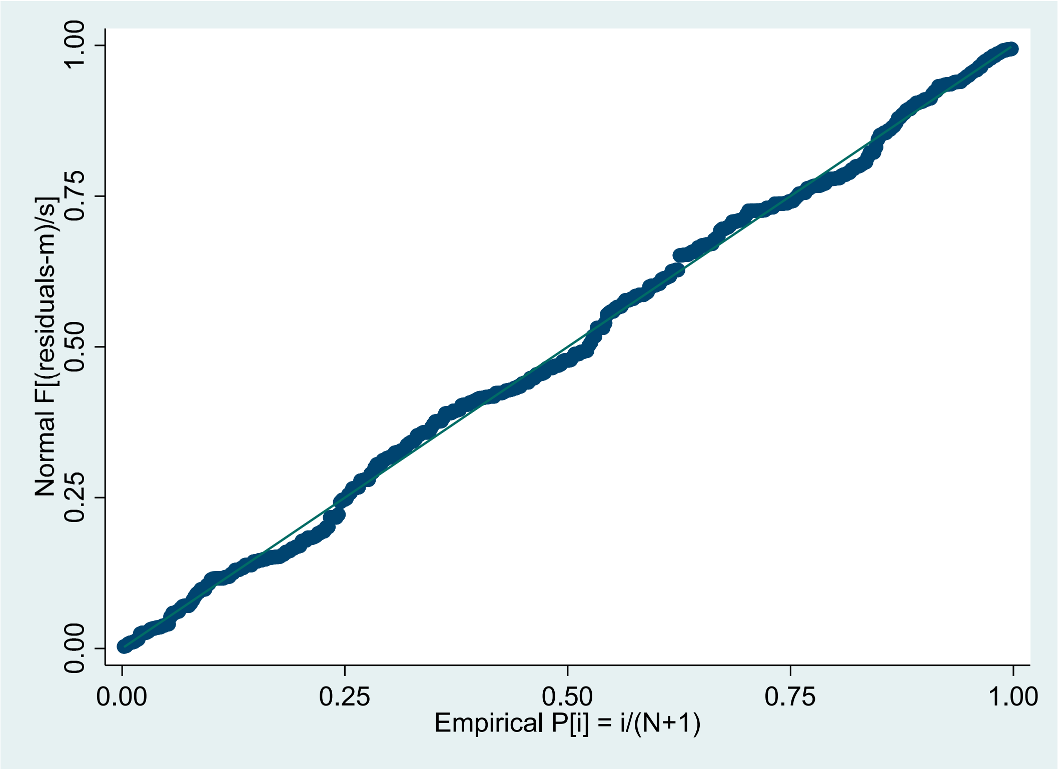

30'

```
------------------------------------------------------
             |            Delta-method    Bonferroni
             |   Contrast   std. err.      z    P>|z|
-------------+----------------------------------------
        dose |
    1 vs 0   |  -.5706312   .3551603    -1.61   1.000
    2 vs 0   |  -2.077452   .3547661    -5.86   0.000
```

```
     3 vs 0  |  -3.888595   .3548647   -10.96   0.000
     4 vs 0  |  -4.815013   .3548647   -13.57   0.000
     2 vs 1  |  -1.506821   .3551603    -4.24   0.000
     3 vs 1  |  -3.317964   .3548647    -9.35   0.000
     4 vs 1  |  -4.244382   .3556524   -11.93   0.000
     3 vs 2  |  -1.811143   .3548647    -5.10   0.000
     4 vs 2  |  -2.737561   .3548647    -7.71   0.000
     4 vs 3  |  -.9264181   .3551603    -2.61   0.091
-----------------------------------------------------
```

60’

```
-----------------------------------------------------
             |            Delta-method    Bonferroni
             |   Contrast   std. err.      z    P>|z|
-------------+---------------------------------------
        dose |
     1 vs 0  |  -1.297771   .4246828    -3.06   0.022
     2 vs 0  |  -4.083464   .4243532    -9.62   0.000
     3 vs 0  |   -6.31849   .4244357   -14.89   0.000
     4 vs 0  |  -7.687136   .4244357   -18.11   0.000
     2 vs 1  |  -2.785693   .4246828    -6.56   0.000
     3 vs 1  |  -5.020719   .4244357   -11.83   0.000
     4 vs 1  |  -6.389365   .4250945   -15.03   0.000
     3 vs 2  |  -2.235026   .4244357    -5.27   0.000
     4 vs 2  |  -3.603672   .4244357    -8.49   0.000
     4 vs 3  |  -1.368646   .4246828    -3.22   0.013
-----------------------------------------------------
```

90’

```
-----------------------------------------------------
             |            Delta-method    Bonferroni
             |   Contrast   std. err.      z    P>|z|
-------------+---------------------------------------
        dose |
     1 vs 0  |  -1.239973   .3501825    -3.54   0.004
     2 vs 0  |  -4.713623   .3497827   -13.48   0.000
     3 vs 0  |  -6.758901   .3498827   -19.32   0.000
     4 vs 0  |  -8.421906   .3498827   -24.07   0.000
     2 vs 1  |   -3.47365   .3501825    -9.92   0.000
     3 vs 1  |  -5.518928   .3498827   -15.77   0.000
```

	Contrast	Delta-method std. err.	z	Bonferroni P>|z|
4 vs 1	-7.181933	.3506816	-20.48	0.000
3 vs 2	-2.045278	.3498827	-5.85	0.000
4 vs 2	-3.708283	.3498827	-10.60	0.000
4 vs 3	-1.663005	.3501825	-4.75	0.000

120’

	Contrast	Delta-method std. err.	z	Bonferroni P>|z|
dose				
1 vs 0	-.584459	.4331473	-1.35	1.000
2 vs 0	-3.533384	.4328241	-8.16	0.000
3 vs 0	-5.435066	.4329049	-12.55	0.000
4 vs 0	-7.130357	.4329049	-16.47	0.000
2 vs 1	-2.948925	.4331473	-6.81	0.000
3 vs 1	-4.850607	.4329049	-11.20	0.000
4 vs 1	-6.545898	.4335509	-15.10	0.000
3 vs 2	-1.901682	.4329049	-4.39	0.000
4 vs 2	-3.596973	.4329049	-8.31	0.000
4 vs 3	-1.695291	.4331473	-3.91	0.001

180’

	Contrast	Delta-method std. err.	z	Bonferroni P>|z|
dose				
1 vs 0	.7713075	.4331473	1.78	0.750
2 vs 0	1.528769	.4328241	3.53	0.004
3 vs 0	-.7667566	.4329049	-1.77	0.765
4 vs 0	-1.711405	.4329049	-3.95	0.001
2 vs 1	.7574618	.4331473	1.75	0.803
3 vs 1	-1.538064	.4329049	-3.55	0.004
4 vs 1	-2.482713	.4335509	-5.73	0.000
3 vs 2	-2.295526	.4329049	-5.30	0.000
4 vs 2	-3.240175	.4329049	-7.48	0.000
4 vs 3	-.9446487	.4331473	-2.18	0.292

Figure 8. dSWDs

	Coefficient	Std. err.	z	P>|z|	[95% conf.	interval]
dose						
1	-4.805695	5.192424	-0.93	0.355	-14.98266	5.371268
2	-3.999888	5.191055	-0.77	0.441	-14.17417	6.174394
3	-1.078384	5.194581	-0.21	0.836	-11.25958	9.102808
timeknot1	-.131423	.1244766	-1.06	0.291	-.3753927	.1125467
timeknot2	1.317873	1.134608	1.16	0.245	-.9059174	3.541663
timeknot3	-3.905106	3.479842	-1.12	0.262	-10.72547	2.91526
timeknot4	4.430392	4.806446	0.92	0.357	-4.990068	13.85085
timeknot5	-2.196626	4.806445	-0.46	0.648	-11.61708	7.223833
dose#c.timeknot1						
1	-.2187311	.1760365	-1.24	0.214	-.5637564	.1262942
2	-.1621575	.1760365	-0.92	0.357	-.5071827	.1828678
3	.0836232	.1760365	0.48	0.635	-.2614021	.4286484
dose#c.timeknot2						
1	.2353414	1.604577	0.15	0.883	-2.909573	3.380255
2	3.248002	1.604577	2.02	0.043	.1030879	6.392916
3	-1.635003	1.604577	-1.02	0.308	-4.779916	1.509911
dose#c.timeknot3						
1	2.333653	4.92124	0.47	0.635	-7.311801	11.97911
2	-10.65078	4.92124	-2.16	0.030	-20.29624	-1.005329
3	4.391356	4.92124	0.89	0.372	-5.254098	14.03681
dose#c.timeknot4						
1	-8.149409	6.797341	-1.20	0.231	-21.47195	5.173134
2	13.03697	6.797341	1.92	0.055	-.2855691	26.35952
3	-1.650386	6.797341	-0.24	0.808	-14.97293	11.67216
dose#c.timeknot5						

1	9.425291	6.797339	1.39	0.166	-3.897249	22.74783
2	-6.632795	6.797339	-0.98	0.329	-19.95534	6.689746
3	-6.326606	6.797339	-0.93	0.352	-19.64915	6.995934
baseline	.0675929	.0809011	0.84	0.403	-.0909703	.2261561
_cons	102.1248	9.234152	11.06	0.000	84.02623	120.2234

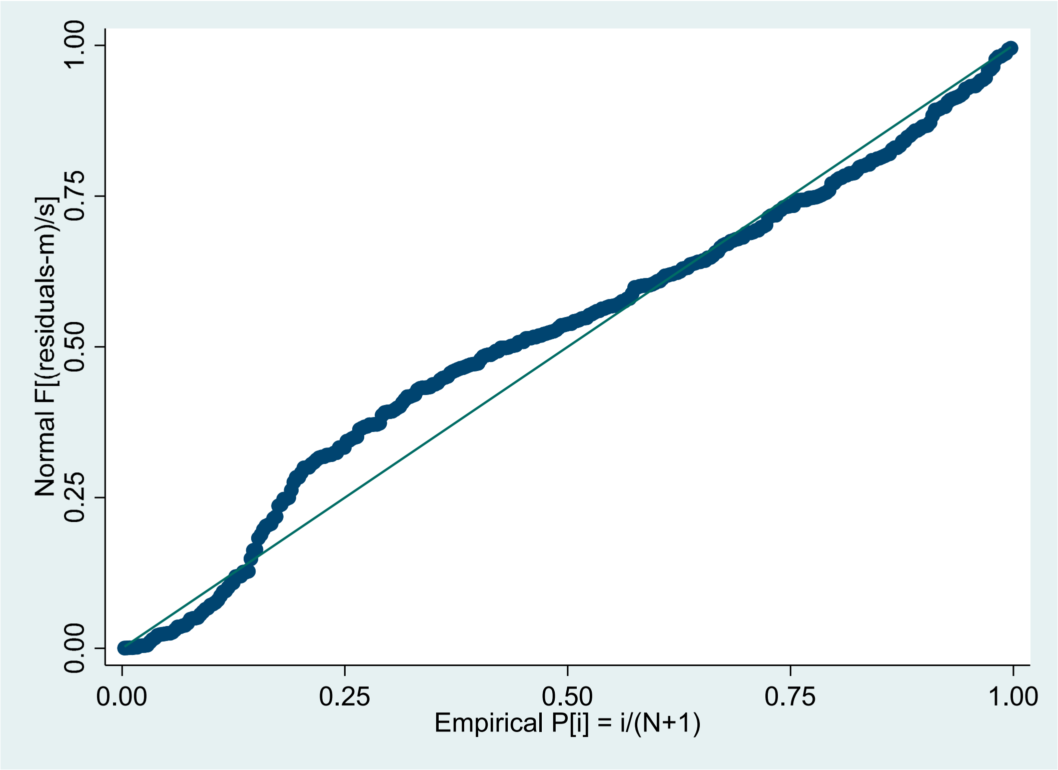

30’

	Contrast	Delta-method std. err.	z	Bonferroni P>|z|
dose				
1 vs 0	-11.29703	3.445344	-3.28	0.006
2 vs 0	-7.890212	3.443281	-2.29	0.132
3 vs 0	.9398103	3.448594	0.27	1.000
2 vs 1	3.406814	3.43785	0.99	1.000
3 vs 1	12.23684	3.437976	3.56	0.002
3 vs 2	8.830022	3.43857	2.57	0.061

.

60’

dose	Contrast	Delta-method std. err.	z	Bonferroni P>|z|
1 vs 0	-17.36474	4.118391	-4.22	0.000
2 vs 0	-5.934132	4.116666	-1.44	0.897
3 vs 0	.0149999	4.12111	0.00	1.000
2 vs 1	11.43061	4.112124	2.78	0.033
3 vs 1	17.37974	4.112229	4.23	0.000
3 vs 2	5.949131	4.112725	1.45	0.888

90’

dose	Contrast	Delta-method std. err.	z	Bonferroni P>|z|
1 vs 0	-21.88513	3.397163	-6.44	0.000
2 vs 0	4.519521	3.395071	1.33	1.000
3 vs 0	-5.478413	3.40046	-1.61	0.643
2 vs 1	26.40465	3.389563	7.79	0.000
3 vs 1	16.40672	3.38969	4.84	0.000
3 vs 2	-9.997934	3.390293	-2.95	0.019

120’

dose	Contrast	Delta-method std. err.	z	Bonferroni P>|z|
1 vs 0	-20.9341	4.200349	-4.98	0.000
2 vs 0	13.34097	4.198657	3.18	0.009
3 vs 0	-11.8964	4.203015	-2.83	0.028
2 vs 1	34.27508	4.194204	8.17	0.000
3 vs 1	9.037705	4.194307	2.15	0.187
3 vs 2	-25.23737	4.194794	-6.02	0.000

180’

dose	Contrast	Delta-method std. err.	z	Bonferroni P>|z|
1 vs 0	-3.679608	4.200349	-0.88	1.000
2 vs 0	4.075992	4.198657	0.97	1.000
3 vs 0	-11.62127	4.203015	-2.76	0.034
2 vs 1	7.7556	4.194204	1.85	0.387
3 vs 1	-7.941663	4.194307	-1.89	0.350
3 vs 2	-15.69726	4.194794	-3.74	0.001

Figure 8. nSWDs

```
------------------------------------------------------------------------------
                 | Coefficient  Std. err.      z    P>|z|     [95% conf. interval]
-----------------+------------------------------------------------------------
            dose |
              1  |   2.364684   .5974023     3.96   0.000     1.193797    3.535571
              2  |  -1.192192   .5977221    -1.99   0.046    -2.363706   -.0206783
              3  |   .1560199   .5977221     0.26   0.794    -1.015494    1.327534
                 |
       timeknot1 |   .0014864   .0143292     0.10   0.917    -.0265984    .0295712
       timeknot2 |  -.0224368   .1306114    -0.17   0.864    -.2784305    .2335569
       timeknot3 |   .0658706   .4005853     0.16   0.869    -.7192623    .8510034
       timeknot4 |  -.0394331   .5532985    -0.07   0.943    -1.123878    1.045012
       timeknot5 |   -.066727   .5532984    -0.12   0.904    -1.151172    1.017718
                 |
dose#c.timeknot1 |
              1  |   -.037023   .0202646    -1.83   0.068    -.0767409    .0026949
              2  |  -.0717016   .0202646    -3.54   0.000    -.1114195   -.0319837
              3  |  -.2197165   .0202646   -10.84   0.000    -.2594344   -.1799986
                 |
dose#c.timeknot2 |
              1  |   .2309098   .1847124     1.25   0.211    -.1311199    .5929396
              2  |   .1387366   .1847124     0.75   0.453    -.2232931    .5007663
              3  |   1.385215   .1847124     7.50   0.000     1.023185    1.747245
                 |
dose#c.timeknot3 |
              1  |   -.532346   .5665132    -0.94   0.347    -1.642691    .5779995
              2  |   .5022199   .5665132     0.89   0.375    -.6081256    1.612565
              3  |  -3.001431   .5665132    -5.30   0.000    -4.111776   -1.891085
                 |
dose#c.timeknot4 |
              1  |   .1734434   .7824823     0.22   0.825    -1.360194     1.70708
              2  |  -2.051956   .7824823    -2.62   0.009    -3.585593   -.5183192
              3  |   1.922871   .7824823     2.46   0.014     .3892337    3.456508
                 |
dose#c.timeknot5 |
```

1	.611137	.7824821	0.78	0.435	-.9224998	2.144774
2	1.95513	.7824821	2.50	0.012	.4214931	3.488767
3	-.8396296	.7824821	-1.07	0.283	-2.373266	.6940072
baseline	-.0748663	.0699457	-1.07	0.284	-.2119573	.0622247
_cons	13.12329	.9473914	13.85	0.000	11.26644	14.98014

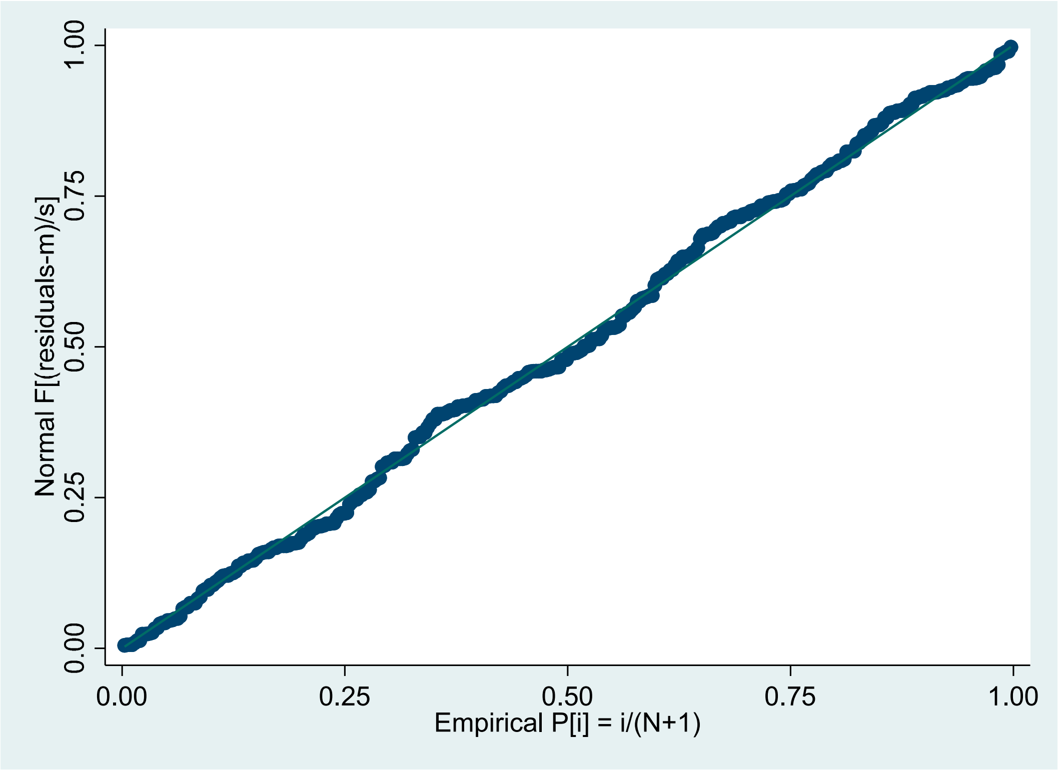

30’

		Delta-method	Bonferroni	
	Contrast	std. err.	z	P>|z|
dose				
1 vs 0	1.323268	.3961188	3.34	0.005
2 vs 0	-3.301618	.396601	-8.32	0.000
3 vs 0	-6.019912	.396601	-15.18	0.000
2 vs 1	-4.624887	.39814	-11.62	0.000
3 vs 1	-7.34318	.39814	-18.44	0.000
3 vs 2	-2.718293	.3957327	-6.87	0.000

.

60’

dose	Contrast	Delta-method std. err.	z	Bonferroni P>|z|
1 vs 0	.6974901	.4736783	1.47	0.845
2 vs 0	-5.161319	.4740815	-10.89	0.000
3 vs 0	-9.702456	.4740815	-20.47	0.000
2 vs 1	-5.858809	.4753698	-12.32	0.000
3 vs 1	-10.39995	.4753698	-21.88	0.000
3 vs 2	-4.541137	.4733554	-9.59	0.000

90’

dose	Contrast	Delta-method std. err.	z	Bonferroni P>|z|
1 vs 0	.7432835	.3905654	1.90	0.342
2 vs 0	-6.370901	.3910544	-16.29	0.000
3 vs 0	-9.298654	.3910544	-23.78	0.000
2 vs 1	-7.114185	.3926152	-18.12	0.000
3 vs 1	-10.04194	.3926152	-25.58	0.000
3 vs 2	-2.927753	.3901738	-7.50	0.000

120’

dose	Contrast	Delta-method std. err.	z	Bonferroni P>|z|
1 vs 0	1.077767	.483121	2.23	0.154
2 vs 0	-5.92731	.4835164	-12.26	0.000
3 vs 0	-6.817266	.4835164	-14.10	0.000
2 vs 1	-7.005078	.4847796	-14.45	0.000
3 vs 1	-7.895033	.4847796	-16.29	0.000
3 vs 2	-.8899556	.4828045	-1.84	0.392

180’

	Contrast	Delta-method std. err.	z	Bonferroni P>|z|
dose				
1 vs 0	.8587294	.483121	1.78	0.453
2 vs 0	-.3904153	.4835164	-0.81	1.000
3 vs 0	-2.64359	.4835164	-5.47	0.000
2 vs 1	-1.249145	.4847795	-2.58	0.060
3 vs 1	-3.502319	.4847795	-7.22	0.000
3 vs 2	-2.253175	.4828044	-4.67	0.000

Figure 9. dSWDs

	Coefficient	Std. err.	z	P>|z|	[95% conf.	interval]
dose						
1	6.327237	4.277689	1.48	0.139	-2.05688	14.71135
2	13.17785	4.253987	3.10	0.002	4.840193	21.51552
3	9.300857	4.251238	2.19	0.029	.968583	17.63313
timeknot1	.0426707	.1000482	0.43	0.670	-.1534202	.2387617
timeknot2	-.1810587	.9119422	-0.20	0.843	-1.968433	1.606315
timeknot3	.0088317	2.796928	0.00	0.997	-5.473046	5.490709
timeknot4	1.413905	3.863186	0.37	0.714	-6.157802	8.985611
timeknot5	-2.727484	3.863186	-0.71	0.480	-10.29919	4.844221
dose#c.timeknot1						
1	.0422242	.1414896	0.30	0.765	-.2350903	.3195387
2	.1999998	.1414896	1.41	0.157	-.0773146	.4773143
3	.1235055	.1414896	0.87	0.383	-.153809	.4008199
dose#c.timeknot2						
1	-1.001879	1.289681	-0.78	0.437	-3.529608	1.525849
2	-2.036923	1.289681	-1.58	0.114	-4.564651	.4908054
3	-1.10319	1.289681	-0.86	0.392	-3.630918	1.424538
dose#c.timeknot3						
1	3.807644	3.955453	0.96	0.336	-3.944901	11.56019
2	6.533911	3.955453	1.65	0.099	-1.218634	14.28646
3	3.731729	3.955453	0.94	0.345	-4.020816	11.48427
dose#c.timeknot4						
1	-5.990418	5.463371	-1.10	0.273	-16.69843	4.717591
2	-9.960113	5.463371	-1.82	0.068	-20.66812	.7478973
3	-6.64745	5.463371	-1.22	0.224	-17.35546	4.06056
dose#c.timeknot5						

1	4.661036	5.46337	0.85	0.394	-6.046972	15.36904
2	10.12658	5.46337	1.85	0.064	-.5814269	20.83459
3	8.096992	5.46337	1.48	0.138	-2.611016	18.805
baseline	.0548594	.0850084	0.65	0.519	-.1117541	.2214728
_cons	97.7322	9.447617	10.34	0.000	79.21521	116.2492

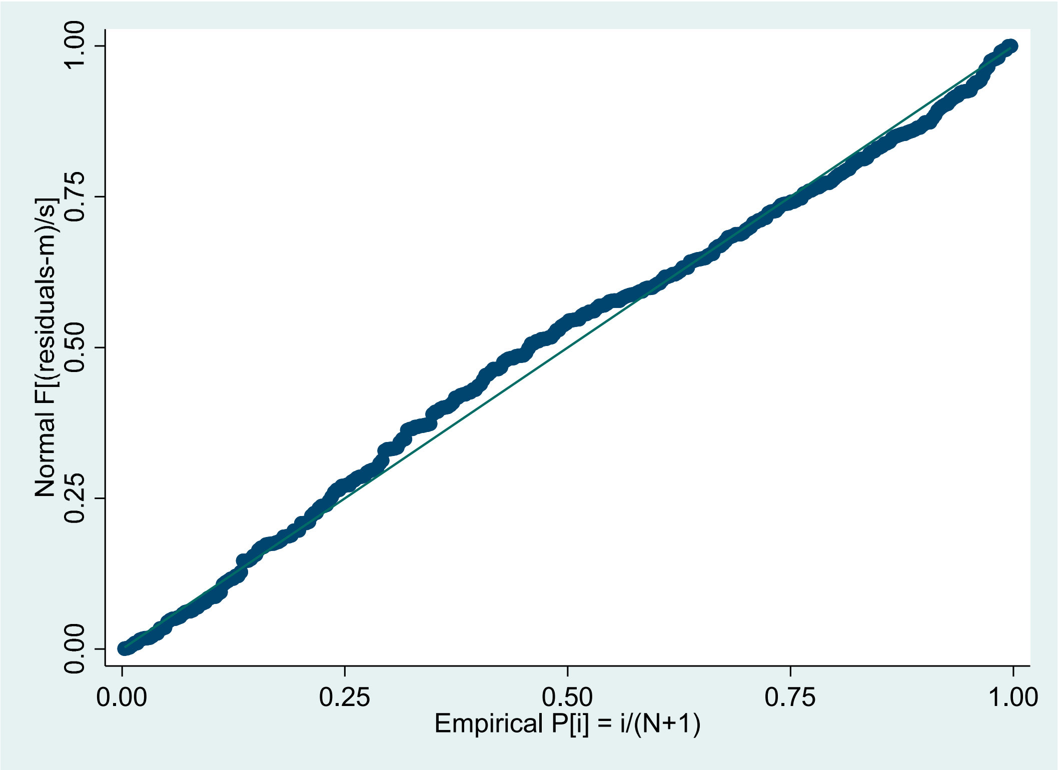

30’

	Contrast	Delta-method std. err.	Bonferroni z	P>|z|
dose				
1 vs 0	7.293398	2.923984	2.49	0.076
2 vs 0	18.56677	2.889198	6.43	0.000
3 vs 0	12.67506	2.885149	4.39	0.000
2 vs 1	11.27337	2.901832	3.88	0.001
3 vs 1	5.381666	2.915457	1.85	0.389
3 vs 2	-5.891709	2.886616	-2.04	0.247

60’

dose	Contrast	Delta-method std. err.	z	Bonferroni P>|z|
1 vs 0	6.456178	3.440695	1.88	0.364
2 vs 0	20.28923	3.411183	5.95	0.000
3 vs 0	14.06353	3.407754	4.13	0.000
2 vs 1	13.83305	3.421891	4.04	0.000
3 vs 1	7.607351	3.433452	2.22	0.160
3 vs 2	-6.225702	3.408996	-1.83	0.407

90’

dose	Contrast	Delta-method std. err.	z	Bonferroni P>|z|
1 vs 0	3.154485	2.887335	1.09	1.000
2 vs 0	16.63894	2.852102	5.83	0.000
3 vs 0	12.60003	2.848001	4.42	0.000
2 vs 1	13.48445	2.8649	4.71	0.000
3 vs 1	9.445542	2.8787	3.28	0.006
3 vs 2	-4.03891	2.849486	-1.42	0.938

120’

dose	Contrast	Delta-method std. err.	z	Bonferroni P>|z|
1 vs 0	1.296404	3.504116	0.37	1.000
2 vs 0	13.7503	3.475142	3.96	0.000
3 vs 0	11.89641	3.471777	3.43	0.004
2 vs 1	12.4539	3.485654	3.57	0.002
3 vs 1	10.60001	3.497005	3.03	0.015
3 vs 2	-1.85389	3.472996	-0.53	1.000

180’

dose	Contrast	Delta-method std. err.	z	Bonferroni P>|z|
1 vs 0	7.735584	3.504116	2.21	0.164
2 vs 0	18.73204	3.475142	5.39	0.000
3 vs 0	15.74044	3.471777	4.53	0.000
2 vs 1	10.99646	3.485653	3.15	0.010
3 vs 1	8.004852	3.497004	2.29	0.132
3 vs 2	-2.991607	3.472996	-0.86	1.000

Figure 9. nSWDs

	Coefficient	Std. err.	z	P>|z|	[95% conf.	interval]
dose						
1	-.5646768	.6605432	-0.85	0.393	-1.859318	.7299641
2	.8829244	.6572254	1.34	0.179	-.4052138	2.171063
3	.4552775	.6572254	0.69	0.488	-.8328607	1.743416
timeknot1	.0083207	.0157069	0.53	0.596	-.0224643	.0391057
timeknot2	-.1030144	.1431691	-0.72	0.472	-.3836206	.1775919
timeknot3	.3323874	.4390997	0.76	0.449	-.5282322	1.193007
timeknot4	-.5130361	.6064956	-0.85	0.398	-1.701746	.6756733
timeknot5	.6382634	.6064954	1.05	0.293	-.5504458	1.826973
dose#c.timeknot1						
1	.0178013	.022213	0.80	0.423	-.0257353	.0613379
2	-.0027699	.022213	-0.12	0.901	-.0463065	.0407667
3	.014114	.022213	0.64	0.525	-.0294226	.0576506
dose#c.timeknot2						
1	-.1754896	.2024717	-0.87	0.386	-.5723268	.2213476
2	.0904951	.2024717	0.45	0.655	-.3063421	.4873322
3	-.0181085	.2024717	-0.09	0.929	-.4149457	.3787287
dose#c.timeknot3						
1	.4959327	.6209808	0.80	0.425	-.7211673	1.713033
2	-.3683754	.6209808	-0.59	0.553	-1.585475	.8487246
3	-.1692193	.6209808	-0.27	0.785	-1.386319	1.047881
dose#c.timeknot4						
1	-.3992784	.8577142	-0.47	0.642	-2.080367	1.281811
2	.7348673	.8577142	0.86	0.392	-.9462218	2.415956
3	.7678771	.8577142	0.90	0.371	-.9132119	2.448966
dose#c.timeknot5						

1	-.2412654	.8577141	-0.28	0.778	-1.922354	1.439823
2	-1.160734	.8577141	-1.35	0.176	-2.841823	.5203547
3	-1.36169	.8577141	-1.59	0.112	-3.042779	.3193985
baseline	-.0354545	.0591408	-0.60	0.549	-.1513684	.0804593
_cons	12.89556	.8226703	15.68	0.000	11.28315	14.50796

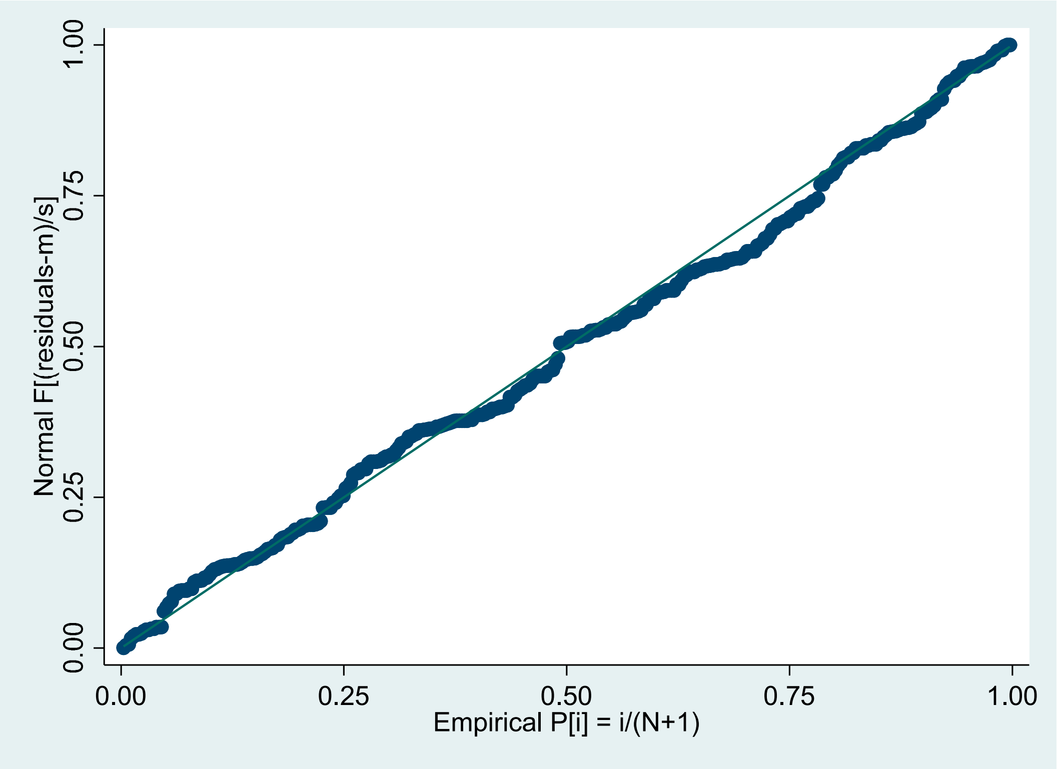

30'

		Delta-method		Bonferroni
	Contrast	std. err.	z	P>|z|
dose				
1 vs 0	-.0832856	.4427586	-0.19	1.000
2 vs 0	.8269763	.4377935	1.89	0.353
3 vs 0	.8732645	.4377935	1.99	0.276
2 vs 1	.9102619	.4347872	2.09	0.218
3 vs 1	.9565501	.4347872	2.20	0.167
3 vs 2	.0462882	.4337805	0.11	1.000

60’

dose	Contrast	Delta-method std. err.	z	Bonferroni P>|z|
1 vs 0	.0822243	.5263952	0.16	1.000
2 vs 0	.9339194	.5222259	1.79	0.442
3 vs 0	1.258656	.5222259	2.41	0.096
2 vs 1	.8516951	.5197082	1.64	0.608
3 vs 1	1.176432	.5197082	2.26	0.142
3 vs 2	.3247368	.5188663	0.63	1.000

90’

dose	Contrast	Delta-method std. err.	z	Bonferroni P>|z|
1 vs 0	-.2352486	.4367905	-0.54	1.000
2 vs 0	1.256132	.4317568	2.91	0.022
3 vs 0	1.528092	.4317568	3.54	0.002
2 vs 1	1.491381	.4287082	3.48	0.003
3 vs 1	1.76334	.4287082	4.11	0.000
3 vs 2	.2719595	.4276871	0.64	1.000

120’

dose	Contrast	Delta-method std. err.	z	Bonferroni P>|z|
1 vs 0	-.6076864	.5366074	-1.13	1.000
2 vs 0	1.403942	.5325181	2.64	0.050
3 vs 0	1.395146	.5325181	2.62	0.053
2 vs 1	2.011629	.5300493	3.80	0.001
3 vs 1	2.002833	.5300493	3.78	0.001
3 vs 2	-.0087961	.5292238	-0.02	1.000

180’

	Contrast	Delta-method std. err.	z	Bonferroni P>|z|
dose				
1 vs 0	-.1685349	.5366074	-0.31	1.000
2 vs 0	.9392989	.532518	1.76	0.467
3 vs 0	.416259	.532518	0.78	1.000
2 vs 1	1.107834	.5300493	2.09	0.220
3 vs 1	.5847939	.5300493	1.10	1.000
3 vs 2	-.5230399	.5292238	-0.99	1.000

Figure 10. dSWDs

```
------------------------------------------------------------------------------
                 | Coefficient  Std. err.      z    P>|z|     [95% conf. interval]
-----------------+------------------------------------------------------------
            dose |
              1  |   2.946703   4.374672     0.67   0.501    -5.627496     11.5209
              2  |   5.303087   4.357845     1.22   0.224    -3.238132     13.8443
              3  |   1.424236    4.38436     0.32   0.745    -7.168953    10.01742
                 |
       timeknot1 |  -.0908023   .1045681    -0.87   0.385     -.295752    .1141473
       timeknot2 |   .9291245   .9531406     0.97   0.330    -.9389968    2.797246
       timeknot3 |  -2.680256   2.923283    -0.92   0.359    -8.409786    3.049274
       timeknot4 |   2.239151   4.037712     0.55   0.579    -5.674619    10.15292
       timeknot5 |     1.3107   4.037711     0.32   0.745    -6.603069    9.224469
                 |
dose#c.timeknot1 |
              1  |  -.2077389   .1478816    -1.40   0.160    -.4975815    .0821037
              2  |  -.3894524   .1478816    -2.63   0.008     -.679295   -.0996098
              3  |  -.7663529   .1478816    -5.18   0.000    -1.056195   -.4765103
                 |
dose#c.timeknot2 |
              1  |  -3.216806   1.347944    -2.39   0.017    -5.858728   -.5748834
              2  |  -3.439261   1.347944    -2.55   0.011    -6.081183   -.7973382
              3  |  -.1127111   1.347944    -0.08   0.933    -2.754634    2.529211
                 |
dose#c.timeknot3 |
              1  |   16.49216   4.134147     3.99   0.000     8.389381    24.59494
              2  |   20.12789   4.134147     4.87   0.000     12.02511    28.23067
              3  |   10.25274   4.134147     2.48   0.013     2.149962    18.35552
                 |
dose#c.timeknot4 |
              1  |    -29.683   5.710187    -5.20   0.000    -40.87476   -18.49124
              2  |  -39.98473   5.710187    -7.00   0.000     -51.1765   -28.79297
              3  |  -26.59469   5.710187    -4.66   0.000    -37.78645   -15.40293
                 |
dose#c.timeknot5 |
```

1	22.76216	5.710186	3.99	0.000	11.5704	33.95392
2	34.87015	5.710186	6.11	0.000	23.67839	46.06191
3	22.43915	5.710186	3.93	0.000	11.24739	33.63091
baseline	-.0086992	.0606389	-0.14	0.886	-.1275492	.1101508
_cons	109.7255	7.040773	15.58	0.000	95.92582	123.5251

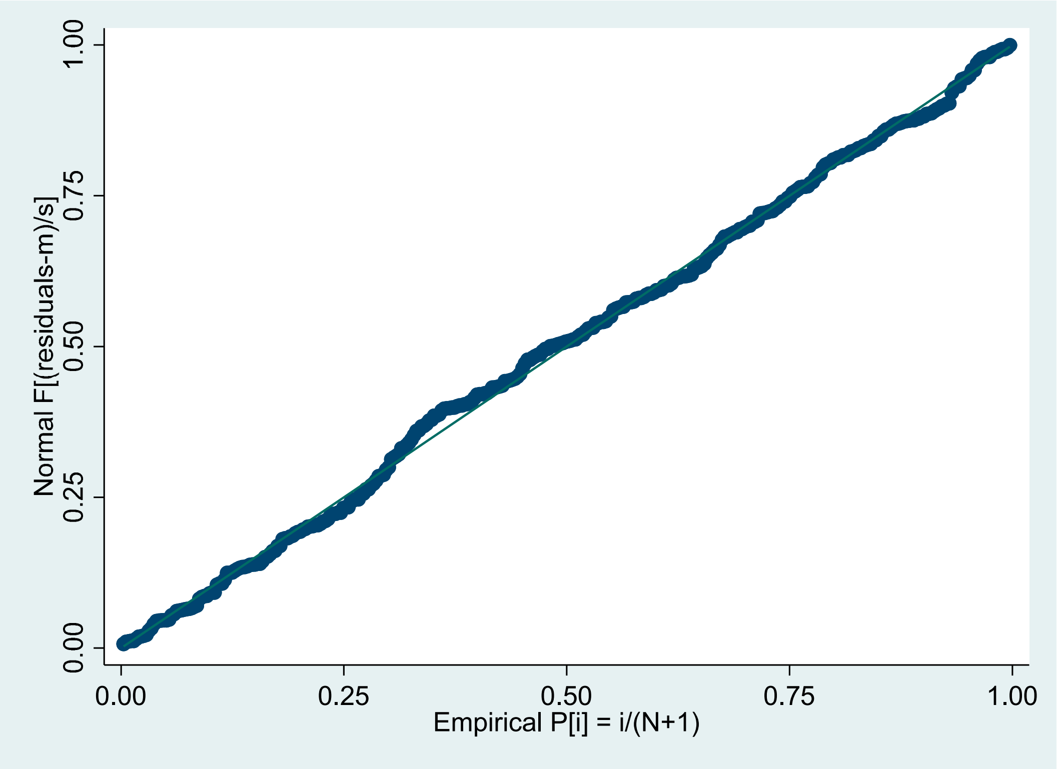

30’

	Contrast	Delta-method std. err.	z	Bonferroni P>|z|
dose				
1 vs 0	-4.250505	2.913429	-1.46	0.867
2 vs 0	-7.412264	2.8881	-2.57	0.062
3 vs 0	-21.60016	2.927956	-7.38	0.000
2 vs 1	-3.161759	2.908843	-1.09	1.000
3 vs 1	-17.34966	2.889526	-6.00	0.000
3 vs 2	-14.1879	2.922177	-4.86	0.000

60’

dose	Contrast	Delta-method std. err.	z	Bonferroni P>|z|
1 vs 0	-17.23796	3.47572	-4.96	0.000
2 vs 0	-26.31828	3.454516	-7.62	0.000
3 vs 0	-44.82744	3.487906	-12.85	0.000
2 vs 1	-9.080321	3.471877	-2.62	0.053
3 vs 1	-27.58948	3.455708	-7.98	0.000
3 vs 2	-18.50916	3.483056	-5.31	0.000

90’

dose	Contrast	Delta-method std. err.	z	Bonferroni P>|z|
1 vs 0	-36.85828	2.873223	-12.83	0.000
2 vs 0	-51.56728	2.847537	-18.11	0.000
3 vs 0	-65.38466	2.887953	-22.64	0.000
2 vs 1	-14.709	2.868574	-5.13	0.000
3 vs 1	-28.52638	2.848983	-10.01	0.000
3 vs 2	-13.81738	2.882094	-4.79	0.000

120’

dose	Contrast	Delta-method std. err.	z	Bonferroni P>|z|
1 vs 0	-44.16345	3.544258	-12.46	0.000
2 vs 0	-59.15807	3.523467	-16.79	0.000
3 vs 0	-68.09559	3.55621	-19.15	0.000
2 vs 1	-14.99462	3.54049	-4.24	0.000

3 vs 1	-23.93213	3.524636	-6.79	0.000
3 vs 2	-8.937512	3.551453	-2.52	0.071

180’

	Contrast	Delta-method std. err.	z	Bonferroni P>|z|
dose				
1 vs 0	2.514933	3.544258	0.71	1.000
2 vs 0	2.829675	3.523467	0.80	1.000
3 vs 0	-10.79759	3.55621	-3.04	0.014
2 vs 1	.3147427	3.54049	0.09	1.000
3 vs 1	-13.31253	3.524636	-3.78	0.001
3 vs 2	-13.62727	3.551453	-3.84	0.001

Figure 10. nSWDs

	Coefficient	Std. err.	z	P>|z|	[95% conf.	interval]
dose						
1	-.7026878	.560155	-1.25	0.210	-1.800571	.3951958
2	-.9249763	.560155	-1.65	0.099	-2.02286	.1729073
3	-.0268976	.5596527	-0.05	0.962	-1.123797	1.070001
timeknot1	-.0013696	.0131804	-0.10	0.917	-.0272028	.0244635
timeknot2	.0523724	.1201401	0.44	0.663	-.1830978	.2878425
timeknot3	-.2266903	.3684696	-0.62	0.538	-.9488775	.495497
timeknot4	.3812019	.5089395	0.75	0.454	-.6163012	1.378705
timeknot5	-.2743449	.5089394	-0.54	0.590	-1.271848	.723158
dose#c.timeknot1						
1	-.0847212	.01864	-4.55	0.000	-.1212549	-.0481876
2	-.1363515	.01864	-7.32	0.000	-.1728851	-.0998178
3	-.2198872	.01864	-11.80	0.000	-.2564209	-.1833536
dose#c.timeknot2						
1	.5708421	.1699037	3.36	0.001	.237837	.9038472
2	.7777785	.1699037	4.58	0.000	.4447734	1.110784
3	1.35412	.1699037	7.97	0.000	1.021114	1.687125
dose#c.timeknot3						
1	-1.070831	.5210948	-2.05	0.040	-2.092158	-.0495038
2	-1.310072	.5210948	-2.51	0.012	-2.331399	-.2887452
3	-2.851814	.5210948	-5.47	0.000	-3.873141	-1.830487
dose#c.timeknot4						
1	.0381417	.7197492	0.05	0.958	-1.372541	1.448824
2	-.1022441	.7197492	-0.14	0.887	-1.512927	1.308438
3	1.685723	.7197492	2.34	0.019	.2750402	3.096405

dose#c.timeknot5						
1	.9740429	.719749	1.35	0.176	-.4366393	2.384725
2	.8217314	.719749	1.14	0.254	-.5889508	2.232414
3	-.7735347	.719749	-1.07	0.282	-2.184217	.6371475
baseline	.0273705	.0848509	0.32	0.747	-.1389342	.1936751
_cons	12.15622	1.112107	10.93	0.000	9.976527	14.33591

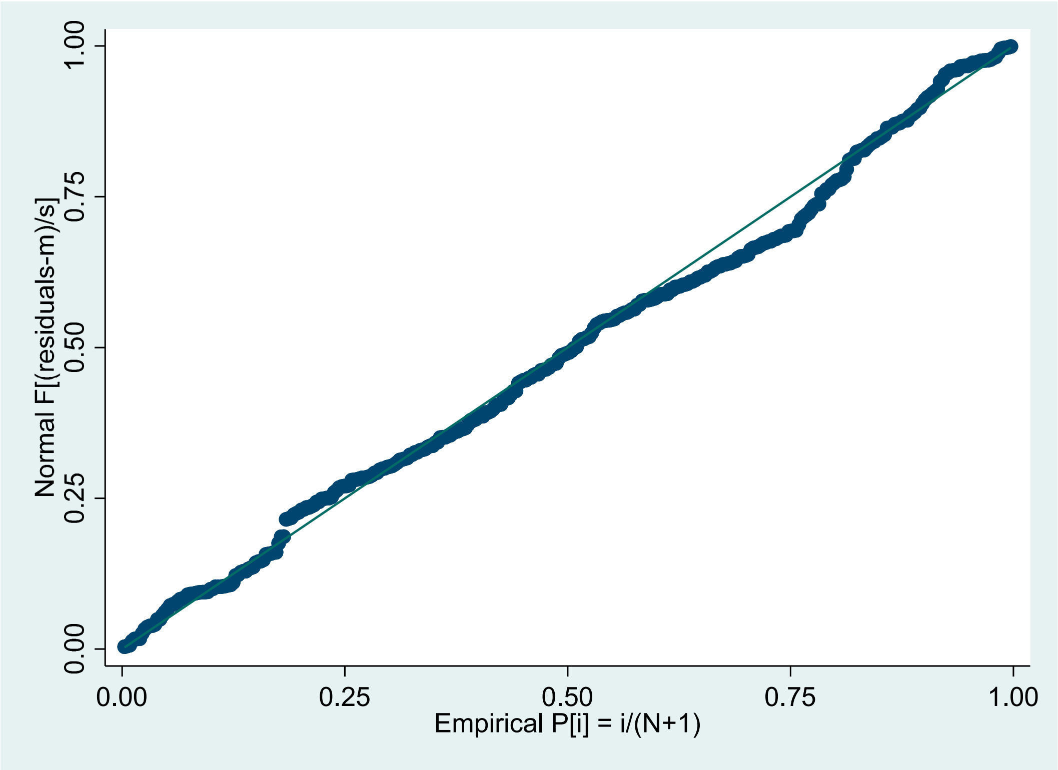

30’

	Contrast	Delta-method std. err.	Bonferroni z	P>|z|
dose				
1 vs 0	-3.073072	.3802293	-8.08	0.000
2 vs 0	-4.782187	.3802293	-12.58	0.000
3 vs 0	-6.217278	.3794889	-16.38	0.000
2 vs 1	-1.709116	.3788956	-4.51	0.000
3 vs 1	-3.144206	.379044	-8.30	0.000
3 vs 2	-1.435091	.379044	-3.79	0.001

60’

dose	Contrast	Delta-method std. err.	z	Bonferroni P>|z|
1 vs 0	-4.41594	.4490567	-9.83	0.000
2 vs 0	-7.239397	.4490567	-16.12	0.000
3 vs 0	-9.970243	.44843	-22.23	0.000
2 vs 1	-2.823457	.447928	-6.30	0.000
3 vs 1	-5.554303	.4480536	-12.40	0.000
3 vs 2	-2.730846	.4480536	-6.09	0.000

90’

dose	Contrast	Delta-method std. err.	z	Bonferroni P>|z|
1 vs 0	-4.025026	.3753371	-10.72	0.000
2 vs 0	-7.289626	.3753371	-19.42	0.000
3 vs 0	-9.703922	.3745871	-25.91	0.000
2 vs 1	-3.2646	.373986	-8.73	0.000
3 vs 1	-5.678896	.3741364	-15.18	0.000
3 vs 2	-2.414296	.3741364	-6.45	0.000

120’

dose	Contrast	Delta-method std. err.	z	Bonferroni P>|z|
1 vs 0	-2.479059	.457489	-5.42	0.000
2 vs 0	-5.49798	.457489	-12.02	0.000
3 vs 0	-7.25862	.4568738	-15.89	0.000

2 vs 1	-3.018921	.4563811	-6.61	0.000
3 vs 1	-4.779561	.4565043	-10.47	0.000
3 vs 2	-1.76064	.4565043	-3.86	0.001

180’

		Delta-method		Bonferroni
	Contrast	std. err.	z	P>|z|
dose				
1 vs 0	.5696519	.457489	1.25	1.000
2 vs 0	-.4669662	.457489	-1.02	1.000
3 vs 0	-2.568739	.4568738	-5.62	0.000
2 vs 1	-1.036618	.4563811	-2.27	0.139
3 vs 1	-3.138391	.4565043	-6.87	0.000
3 vs 2	-2.101773	.4565043	-4.60	0.000

Figure 11. dSWDs

```
------------------------------------------------------------------------------
                 | Coefficient  Std. err.      z    P>|z|     [95% conf. interval]
-----------------+----------------------------------------------------------------
            dose |
              1  |   .4450147   3.598795     0.12   0.902    -6.608494    7.498524
              2  |    6.11919   3.583862     1.71   0.088    -.9050509    13.14343
              3  |   4.719592   3.582456     1.32   0.188    -2.301894    11.74108
                 |
       timeknot1 |  -.0044231   .0857238    -0.05   0.959    -.1724388    .1635925
       timeknot2 |   .1911349    .781375     0.24   0.807    -1.340332    1.722602
       timeknot3 |  -.5954936   2.396478    -0.25   0.804    -5.292504    4.101516
       timeknot4 |   .8207698   3.310075     0.25   0.804    -5.666858    7.308398
       timeknot5 |  -.9864987   3.310075    -0.30   0.766    -7.474126    5.501128
                 |
dose#c.timeknot1 |
              1  |  -.1908524   .1212318    -1.57   0.115    -.4284624    .0467576
              2  |   .0147066   .1212318     0.12   0.903    -.2229034    .2523166
              3  |  -.2865766   .1212318    -2.36   0.018    -.5241866   -.0489666
                 |
dose#c.timeknot2 |
              1  |   -.429172   1.105031    -0.39   0.698    -2.594993    1.736649
              2  |  -6.984897   1.105031    -6.32   0.000    -9.150719   -4.819076
              3  |  -4.745276   1.105031    -4.29   0.000    -6.911098   -2.579455
                 |
dose#c.timeknot3 |
              1  |   2.681295   3.389131     0.79   0.429     -3.96128     9.32387
              2  |   25.72382   3.389131     7.59   0.000     19.08124    32.36639
              3  |   19.76678   3.389131     5.83   0.000      13.1242    26.40936
                 |
dose#c.timeknot4 |
              1  |  -2.977631   4.681153    -0.64   0.525    -12.15252     6.19726
              2  |  -34.19537   4.681153    -7.30   0.000    -43.37026   -25.02048
              3  |  -27.69782   4.681153    -5.92   0.000    -36.87271   -18.52293
                 |
dose#c.timeknot5 |
```

1	-2.201721	4.681152	-0.47	0.638	-11.37661	6.973169
2	18.60893	4.681152	3.98	0.000	9.434038	27.78382
3	13.56916	4.681152	2.90	0.004	4.394269	22.74405
baseline	.0376066	.0538034	0.70	0.485	-.0678462	.1430594
_cons	102.0754	6.183487	16.51	0.000	89.95597	114.1948

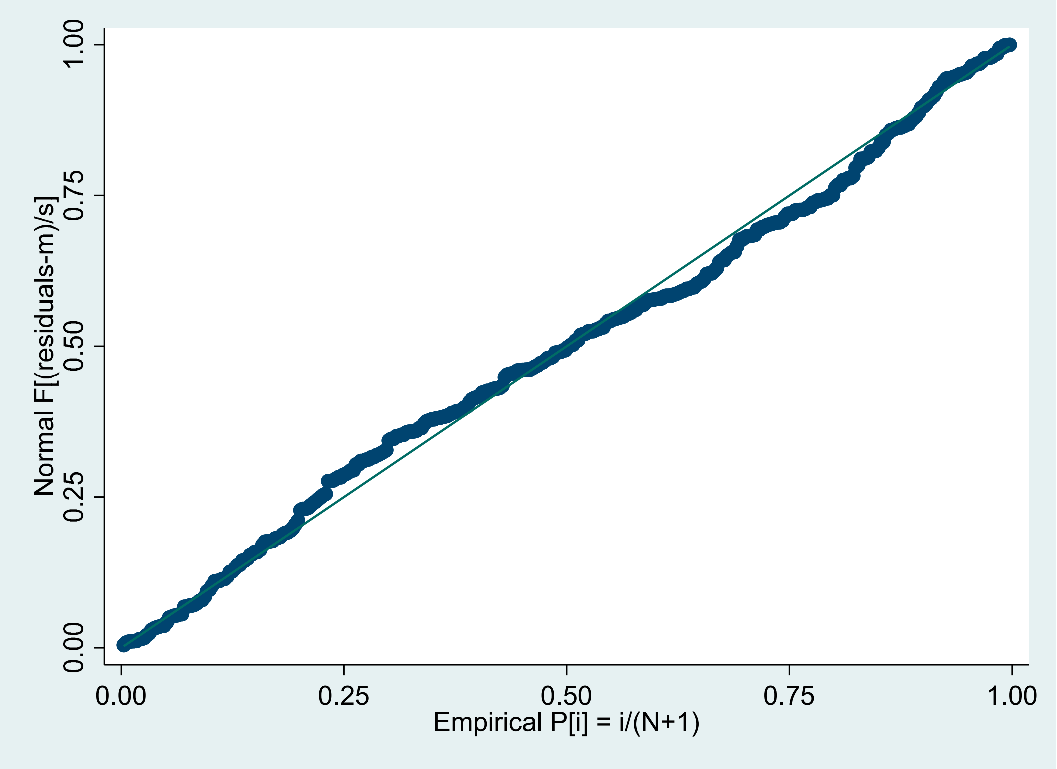

30’

	Contrast	Delta-method std. err.	z	Bonferroni P>|z|
dose				
1 vs 0	-5.409308	2.407104	-2.25	0.148
2 vs 0	4.46492	2.38472	1.87	0.367
3 vs 0	-5.30129	2.382607	-2.22	0.156
2 vs 1	9.874227	2.372108	4.16	0.000
3 vs 1	.1080181	2.373315	0.05	1.000
3 vs 2	-9.766209	2.367517	-4.13	0.000

60’

```
--------------------------------------------------------
             |            Delta-method    Bonferroni
             |   Contrast   std. err.      z    P>|z|
-------------+------------------------------------------
        dose |
     1 vs 0  |  -12.03614   2.865056    -4.20   0.000
     2 vs 0  |  -9.762167   2.846276    -3.43   0.004
     3 vs 0  |  -23.86367   2.844506    -8.39   0.000
     2 vs 1  |   2.273973   2.835718     0.80   1.000
     3 vs 1  |  -11.82753   2.836728    -4.17   0.000
     3 vs 2  |   -14.1015   2.831878    -4.98   0.000
--------------------------------------------------------
```

90’

```
--------------------------------------------------------
             |            Delta-method    Bonferroni
             |   Contrast   std. err.      z    P>|z|
-------------+------------------------------------------
        dose |
     1 vs 0  |   -19.4036   2.374403    -8.17   0.000
     2 vs 0  |  -41.41774   2.351708   -17.61   0.000
     3 vs 0  |  -53.57901   2.349565   -22.80   0.000
     2 vs 1  |  -22.01414   2.338919    -9.41   0.000
     3 vs 1  |  -34.17541   2.340143   -14.60   0.000
     3 vs 2  |  -12.16127   2.334262    -5.21   0.000
--------------------------------------------------------
```

120’

```
--------------------------------------------------------
             |            Delta-method    Bonferroni
             |   Contrast   std. err.      z    P>|z|
-------------+------------------------------------------
        dose |
     1 vs 0  |  -24.26226   2.920942    -8.31   0.000
     2 vs 0  |  -64.48888   2.902523   -22.22   0.000
     3 vs 0  |  -73.33864   2.900787   -25.28   0.000
```

2 vs 1	-40.22662	2.89217	-13.91	0.000
3 vs 1	-49.07638	2.89316	-16.96	0.000
3 vs 2	-8.849756	2.888406	-3.06	0.013

180’

	Contrast	Delta-method std. err.	z	Bonferroni P>|z|
dose				
1 vs 0	-17.3842	2.920942	-5.95	0.000
2 vs 0	-32.02653	2.902523	-11.03	0.000
3 vs 0	-41.31071	2.900787	-14.24	0.000
2 vs 1	-14.64233	2.89217	-5.06	0.000
3 vs 1	-23.9265	2.89316	-8.27	0.000
3 vs 2	-9.284173	2.888406	-3.21	0.008

Figure 11. nSWDs

	Coefficient	Std. err.	z	P>|z|	[95% conf.	interval]
dose						
1	.7162469	.4809591	1.49	0.136	-.2264156	1.658909
2	.5157986	.4809591	1.07	0.284	-.4268639	1.458461
3	2.01294	.481072	4.18	0.000	1.070056	2.955824
timeknot1	-.0007253	.0115403	-0.06	0.950	-.0233438	.0218933
timeknot2	.0324524	.10519	0.31	0.758	-.1737162	.2386211
timeknot3	-.1575469	.3226179	-0.49	0.625	-.7898663	.4747725
timeknot4	.3204619	.4456079	0.72	0.472	-.5529136	1.193837
timeknot5	-.320634	.4456078	-0.72	0.472	-1.194009	.5527413
dose#c.timeknot1						
1	-.0142406	.0163204	-0.87	0.383	-.0462281	.0177468
2	-.0145866	.0163204	-0.89	0.371	-.046574	.0174009
3	-.1073187	.0163204	-6.58	0.000	-.1393062	-.0753312
dose#c.timeknot2						
1	-.2654358	.1487612	-1.78	0.074	-.5570023	.0261307
2	-.6949542	.1487612	-4.67	0.000	-.9865207	-.4033877
3	-.1086007	.1487612	-0.73	0.465	-.4001672	.1829658
dose#c.timeknot3						
1	1.202032	.4562506	2.63	0.008	.3077977	2.096267
2	2.754203	.4562506	6.04	0.000	1.859968	3.648438
3	1.292621	.4562506	2.83	0.005	.3983867	2.186856
dose#c.timeknot4						
1	-2.060036	.6301848	-3.27	0.001	-3.295176	-.8248967
2	-3.913172	.6301848	-6.21	0.000	-5.148312	-2.678033
3	-2.487923	.6301848	-3.95	0.000	-3.723063	-1.252784
dose#c.timeknot5						

1	1.773221	.6301846	2.81	0.005	.5380822	3.008361
2	2.223594	.6301846	3.53	0.000	.9884552	3.458734
3	1.284818	.6301846	2.04	0.041	.0496789	2.519957
baseline	.1162856	.048132	2.42	0.016	.0219486	.2106225
_cons	11.03353	.6858731	16.09	0.000	9.689244	12.37782

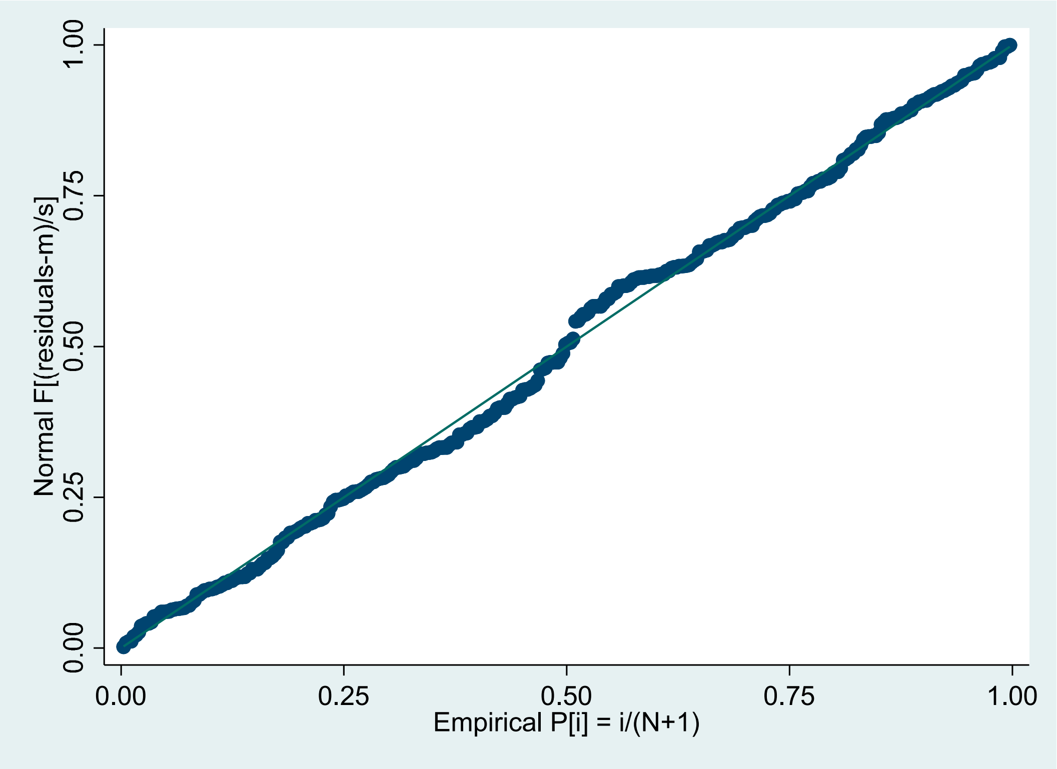

30’

	Contrast	Delta-method std. err.	Bonferroni z	P>|z|
dose				
1 vs 0	.2093975	.3187665	0.66	1.000
2 vs 0	-.1302847	.3187665	-0.41	1.000
3 vs 0	-1.239202	.3189368	-3.89	0.001
2 vs 1	-.3396822	.3187097	-1.07	1.000
3 vs 1	-1.448599	.3192204	-4.54	0.000
3 vs 2	-1.108917	.3192204	-3.47	0.003

60’

dose	Contrast	Delta-method std. err.	z	Bonferroni P>|z|
1 vs 0	-.7752363	.3812719	-2.03	0.252
2 vs 0	-2.027286	.3812719	-5.32	0.000
3 vs 0	-4.686824	.3814143	-12.29	0.000
2 vs 1	-1.252049	.3812244	-3.28	0.006
3 vs 1	-3.911588	.3816515	-10.25	0.000
3 vs 2	-2.659539	.3816515	-6.97	0.000

90’

dose	Contrast	Delta-method std. err.	z	Bonferroni P>|z|
1 vs 0	-2.354829	.3142904	-7.49	0.000
2 vs 0	-5.599861	.3142904	-17.82	0.000
3 vs 0	-8.137623	.3144631	-25.88	0.000
2 vs 1	-3.245032	.3142328	-10.33	0.000
3 vs 1	-5.782794	.3147507	-18.37	0.000
3 vs 2	-2.537762	.3147507	-8.06	0.000

120’

dose	Contrast	Delta-method std. err.	z	Bonferroni P>|z|
1 vs 0	-3.204117	.3888809	-8.24	0.000
2 vs 0	-7.967623	.3888809	-20.49	0.000
3 vs 0	-9.848148	.3890205	-25.32	0.000
2 vs 1	-4.763506	.3888344	-12.25	0.000

3 vs 1	-6.64403	.3892531	-17.07	0.000
3 vs 2	-1.880525	.3892531	-4.83	0.000

180’

	Contrast	Delta-method std. err.	z	Bonferroni P>|z|
dose				
1 vs 0	-.9123686	.3888809	-2.35	0.114
2 vs 0	-3.65373	.3888809	-9.40	0.000
3 vs 0	-5.494439	.3890205	-14.12	0.000
2 vs 1	-2.741361	.3888344	-7.05	0.000
3 vs 1	-4.58207	.3892531	-11.77	0.000
3 vs 2	-1.840709	.3892531	-4.73	0.000

Figure 12. dSWDs

```
------------------------------------------------------------------------------
                 | Coefficient  Std. err.      z    P>|z|     [95% conf. interval]
-----------------+------------------------------------------------------------
            dose |
              1  |  -2.727592   3.077085    -0.89   0.375    -8.758568    3.303385
              2  |   .8654846   3.069759     0.28   0.778    -5.151132    6.882101
              3  |  -4.671747    3.07002    -1.52   0.128    -10.68888    1.345382
                 |
       timeknot1 |   .1130404   .0736203     1.54   0.125    -.0312528    .2573336
       timeknot2 |  -.8035406   .6710511    -1.20   0.231    -2.118777    .5116953
       timeknot3 |   2.211551   2.058114     1.07   0.283    -1.822279    6.245381
       timeknot4 |  -2.677709   2.842719    -0.94   0.346    -8.249336    2.893918
       timeknot5 |   2.491031   2.842719     0.88   0.381    -3.080595    8.062657
                 |
dose#c.timeknot1 |
              1  |  -.3591498   .1041149    -3.45   0.001    -.5632112   -.1550884
              2  |  -1.309529   .1041149   -12.58   0.000     -1.51359   -1.105468
              3  |  -1.589347   .1041149   -15.27   0.000    -1.793409   -1.385286
                 |
dose#c.timeknot2 |
              1  |   1.803101   .9490096     1.90   0.057    -.0569233    3.663126
              2  |   8.323743   .9490096     8.77   0.000     6.463719    10.18377
              3  |   9.827937   .9490096    10.36   0.000     7.967912    11.68796
                 |
dose#c.timeknot3 |
              1  |  -3.385407   2.910613    -1.16   0.245    -9.090104     2.31929
              2  |  -19.72065   2.910613    -6.78   0.000    -25.42535   -14.01595
              3  |  -22.89664   2.910613    -7.87   0.000    -28.60134   -17.19194
                 |
dose#c.timeknot4 |
              1  |   1.528338   4.020212     0.38   0.704    -6.351133    9.407808
              2  |   18.17096   4.020212     4.52   0.000     10.29149    26.05043
              3  |    20.4611   4.020212     5.09   0.000     12.58163    28.34057
                 |
dose#c.timeknot5 |
```

1	-.5593166	4.020211	-0.14	0.889	-8.438786	7.320153
2	-15.18881	4.020211	-3.78	0.000	-23.06828	-7.309345
3	-15.94224	4.020211	-3.97	0.000	-23.8217	-8.062766
baseline	.0434559	.0414717	1.05	0.295	-.0378272	.124739
_cons	84.3573	4.280075	19.71	0.000	75.96851	92.74609

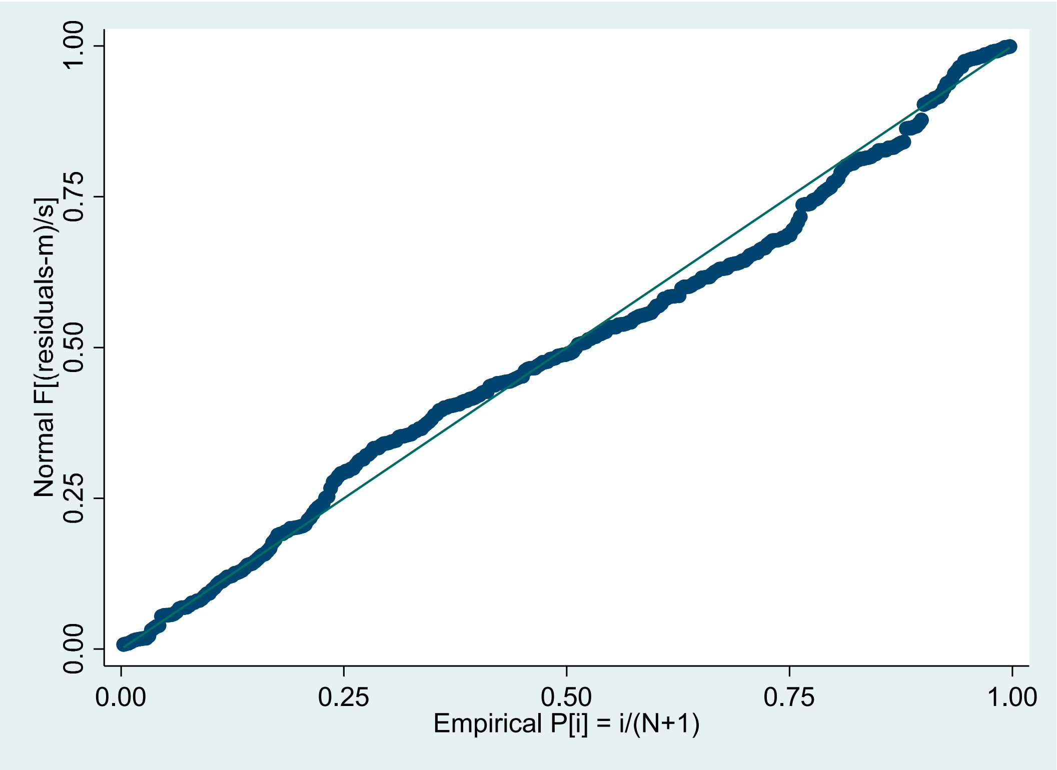

30'

	Contrast	Delta-method std. err.	Bonferroni z	P>|z|
dose				
1 vs 0	-12.96116	2.046868	-6.33	0.000
2 vs 0	-35.92326	2.035837	-17.65	0.000
3 vs 0	-49.40378	2.036231	-24.26	0.000
2 vs 1	-22.96211	2.051264	-11.19	0.000
3 vs 1	-36.44263	2.053453	-17.75	0.000
3 vs 2	-13.48052	2.035548	-6.62	0.000

60’

dose	Contrast	Delta-method std. err.	z	Bonferroni P>|z|
1 vs 0	-19.94914	2.443443	-8.16	0.000
2 vs 0	-57.72927	2.43421	-23.72	0.000
3 vs 0	-76.44553	2.434539	-31.40	0.000
2 vs 1	-37.78014	2.447127	-15.44	0.000
3 vs 1	-56.4964	2.448961	-23.07	0.000
3 vs 2	-18.71626	2.433968	-7.69	0.000

90’

dose	Contrast	Delta-method std. err.	z	Bonferroni P>|z|
1 vs 0	-21.46158	2.018501	-10.63	0.000
2 vs 0	-55.486	2.007315	-27.64	0.000
3 vs 0	-74.9757	2.007714	-37.34	0.000
2 vs 1	-34.02443	2.022959	-16.82	0.000
3 vs 1	-53.51412	2.025178	-26.42	0.000
3 vs 2	-19.4897	2.007021	-9.71	0.000

120’

dose	Contrast	Delta-method std. err.	z	Bonferroni P>|z|
1 vs 0	-19.331	2.491767	-7.76	0.000
2 vs 0	-43.79168	2.482713	-17.64	0.000
3 vs 0	-61.64896	2.483037	-24.83	0.000

2 vs 1	-24.46068	2.495379	-9.80	0.000
3 vs 1	-42.31796	2.497179	-16.95	0.000
3 vs 2	-17.85728	2.482476	-7.19	0.000

180’

	Contrast	Delta-method std. err.	z	Bonferroni P>|z|
dose				
1 vs 0	-11.8654	2.491766	-4.76	0.000
2 vs 0	-30.49729	2.482713	-12.28	0.000
3 vs 0	-44.41277	2.483037	-17.89	0.000
2 vs 1	-18.63189	2.495379	-7.47	0.000
3 vs 1	-32.54736	2.497179	-13.03	0.000
3 vs 2	-13.91547	2.482476	-5.61	0.000

Figure 12. nSWDs

	Coefficient	Std. err.	z	P>|z|	[95% conf.	interval]
dose						
1	1.364356	.5384196	2.53	0.011	.3090733	2.419639
2	-.0513884	.5384196	-0.10	0.924	-1.106671	1.003895
3	-.0889673	.5380627	-0.17	0.869	-1.143551	.9656162
timeknot1	-.0151676	.0129115	-1.17	0.240	-.0404736	.0101384
timeknot2	.1964605	.1176883	1.67	0.095	-.0342043	.4271254
timeknot3	-.5400723	.3609501	-1.50	0.135	-1.247521	.1673769
timeknot4	.4217386	.4985533	0.85	0.398	-.555408	1.398885
timeknot5	.1647279	.4985532	0.33	0.741	-.8124185	1.141874
dose#c.timeknot1						
1	-.034052	.0182596	-1.86	0.062	-.0698401	.0017361
2	-.1365531	.0182596	-7.48	0.000	-.1723412	-.100765
3	-.1789692	.0182596	-9.80	0.000	-.2147573	-.1431811
dose#c.timeknot2						
1	-.0154359	.1664364	-0.09	0.926	-.3416452	.3107734
2	.7785483	.1664364	4.68	0.000	.452339	1.104758
3	.965655	.1664364	5.80	0.000	.6394456	1.291864
dose#c.timeknot3						
1	.3050005	.5104605	0.60	0.550	-.6954837	1.305485
2	-1.624951	.5104605	-3.18	0.001	-2.625436	-.6244672
3	-1.900303	.5104605	-3.72	0.000	-2.900787	-.8998188
dose#c.timeknot4						
1	-.6020817	.7050609	-0.85	0.393	-1.983976	.7798123
2	1.085761	.7050609	1.54	0.124	-.2961326	2.467655
3	.9349571	.7050609	1.33	0.185	-.4469368	2.316851
dose#c.timeknot5						

1	.3006736	.7050608	0.43	0.670	-1.08122	1.682567
2	-.8768236	.7050608	-1.24	0.214	-2.258717	.5050701
3	-.3282438	.7050608	-0.47	0.642	-1.710138	1.05365
baseline	-.0168156	.0522713	-0.32	0.748	-.1192654	.0856343
_cons	12.22068	.7280461	16.79	0.000	10.79373	13.64762

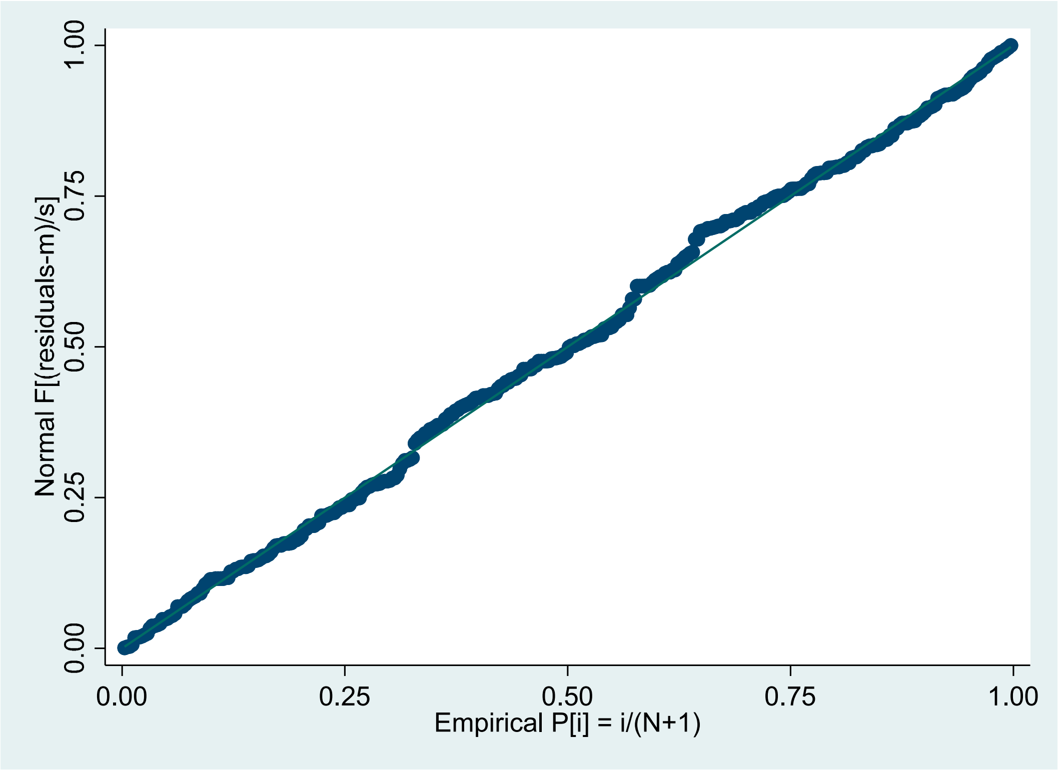

30’

	Contrast	Delta-method std. err.	z	Bonferroni P>|z|
dose				
1 vs 0	.3381658	.3571159	0.95	1.000
2 vs 0	-3.914416	.3571159	-10.96	0.000
3 vs 0	-5.168348	.3565776	-14.49	0.000
2 vs 1	-4.252582	.3587262	-11.85	0.000
3 vs 1	-5.506514	.3571159	-15.42	0.000
3 vs 2	-1.253931	.3571159	-3.51	0.003

60’

dose	Contrast	Delta-method std. err.	Bonferroni z	P>|z|
1 vs 0	-.7158093	.4269702	-1.68	0.562
2 vs 0	-6.376057	.4269702	-14.93	0.000
3 vs 0	-8.509549	.42652	-19.95	0.000
2 vs 1	-5.660248	.4283179	-13.22	0.000
3 vs 1	-7.79374	.4269702	-18.25	0.000
3 vs 2	-2.133492	.4269702	-5.00	0.000

90’

dose	Contrast	Delta-method std. err.	Bonferroni z	P>|z|
1 vs 0	-1.733854	.3521147	-4.92	0.000
2 vs 0	-6.52241	.3521147	-18.52	0.000
3 vs 0	-8.944483	.3515687	-25.44	0.000
2 vs 1	-4.788556	.3537477	-13.54	0.000
3 vs 1	-7.21063	.3521147	-20.48	0.000
3 vs 2	-2.422074	.3521147	-6.88	0.000

120’

dose	Contrast	Delta-method std. err.	Bonferroni z	P>|z|
1 vs 0	-2.286251	.4354756	-5.25	0.000
2 vs 0	-5.389514	.4354756	-12.38	0.000
3 vs 0	-7.585426	.4350342	-17.44	0.000
2 vs 1	-3.103263	.4367971	-7.10	0.000

```
     3 vs 1  |  -5.299175   .4354756   -12.17   0.000
     3 vs 2  |  -2.195912   .4354756    -5.04   0.000
-----------------------------------------------------
```

180’

```
-----------------------------------------------------
             |            Delta-method    Bonferroni
             |   Contrast   std. err.      z    P>|z|
-------------+---------------------------------------
        dose |
     1 vs 0  |  -1.354236   .4354755    -3.11   0.011
     2 vs 0  |  -2.774253   .4354755    -6.37   0.000
     3 vs 0  |  -3.970907   .4350342    -9.13   0.000
     2 vs 1  |  -1.420017    .436797    -3.25   0.007
     3 vs 1  |   -2.61667   .4354755    -6.01   0.000
     3 vs 2  |  -1.196653   .4354755    -2.75   0.036
-----------------------------------------------------
```
